# SINE-derived satellites in scaled reptiles

**DOI:** 10.1186/s13100-023-00309-2

**Published:** 2023-12-07

**Authors:** Nikita S. Vassetzky, Sergei A. Kosushkin, Alexey P. Ryskov

**Affiliations:** 1 Institute of Gene Biology of the Russian Academy of Sciences, Moscow, 119334 Russia; 2 Engelhardt Institute of Molecular Biology, Russian Academy of Sciences, Moscow, 119991 Russia

**Keywords:** Satellite DNA, Tandem repeats, SINEs, Retrotransposons, Squamata, Reptilia, Evolution

## Abstract

**Background:**

The genomes of many eukaryotes contain DNA repeats in the form of both tandem and interspersed elements with distinct structure, evolutionary histories, and mechanisms of emergence and amplification. Although there is considerable knowledge regarding their diversity, there is little evidence directly linking these two types.

**Results:**

Different tandem repeats derived from portions of short interspersed elements (SINEs) belonging to different families were identified in 56 genomes of squamate reptiles. All loci of SINE-derived satellites (sSats) were thoroughly analyzed. Snake sSats exhibited high similarity in both structure and copy number, while other taxa may have highly diverse (geckos), rare (*Darevskia* lizards), or missing sSats (agamid lizards). Similar to most satellites associated with heterochromatin, sSats are likely linked to subtelomeric chromosomal regions.

**Conclusions:**

Discovered tandem repeats derived from SINEs exhibit satellite-like properties, although they have not amplified to the same degree as typical satellites. The autonomous emergence of distinct sSats from diverse SINE families in numerous squamate species suggests a nonrandom process of satellite genesis originating from repetitive SINEs.

**Supplementary Information:**

The online version contains supplementary material available at 10.1186/s13100-023-00309-2.

## Background

Eukaryotic genomes contain two primary types of repetitive elements: tandem repeats and transposable (interspersed) elements. These elements are highly diverse, comprising many classes within a genome and differing in structure, genomic organization, and amplification mechanisms.

Primarily, tandem repeats are organized in long arrays of relatively short non-coding sequences called satellites. Satellites are typically categorized into two or three classes: micro-, mini- and regular satellites. However, sometimes minisatellites are placed in both the micro- and satellite categories. We will follow the division into microsatellites (simple repeats) and proper satellites [1].

Satellite DNAs exhibit variations in nucleotide sequence, sequence complexity, repeat unit length, and abundance. However, they do share two essential features: organization into lengthy arrays of tandem head-to-tail repeats and association with heterochromatic (telomeric or centromeric) regions [2]. The content of satellite DNA varies from 0.5% to more than 50% in animal genomes [1].

Different genomes may contain satellite families specific to certain species, while others may be shared across numerous taxa. However, most satellite families within a species have unrelated sequences. For example, the human genome contains nine satellite families with the predominant α-satellite comprising over half of total satellite DNA [1].

Structural roles of satellite DNA in chromosome organization, pairing, and segregation have been proposed. Telomeric and centromeric regions stabilize the chromatin at these sites for effective interactions with DNA-binding proteins, which is crucial for kinetochore formation and chromosomal segregation during mitosis and meiosis. Subtelomeric satellites stabilize chromosomal ends in a sequence-independent manner [1].

A different type of DNA repeats are transposable elements that comprise two classes: retrotransposons and DNA transposons. Retrotransposons utilize RNA-mediated mechanisms and a copy-and-paste process in their amplification, while DNA transposons rely on DNA-mediated mechanisms and a cut-and-paste process.

Short interspersed elements (SINEs) and long interspersed elements (LINEs) are the most abundant repetitive elements in higher eukaryotes, with SINEs outnumbering LINEs in most vertebrates and plants [3]. SINEs do not encode any proteins and their amplification is dependent on the enzymes of the cell and the partner LINEs. SINEs originate from a limited number of “master” copies, which can vary over time and give rise to numerous subfamilies [4]. SINEs are predominantly found in euchromatin [5].

Certain retrotransposons have distinct evolutionary relationships, such as the 3′-terminal region of a typical SINE originating from its partner LINE. Some SINE families, mostly in mammals, include (TC)_n_ stretches, which resemble microsatellites in both structure and behavior; these structures are hypervariable sites within SINEs [4]. The structure of a SINE typically comprises four distinct regions: a head derived from one of three types of cellular RNAs transcribed by RNA polymerase III (most commonly, tRNA); a body whose origin and function remain largely unknown; a LINE-derived region (LDR) the region necessary for recognition of SINE RNA by the LINE machinery; and a tail composed of a variable-length sequence of simple repeats. This pattern is applicable to three SINE families, Sauria/Squam1 (here referred to as Squam1 to avoid confusion with the Sauria taxonomic name), Squam2, and Squam3, which are relevant to this study [4].

Despite their differing structures and amplification mechanisms, transposable elements and satellites can share similar sequences, suggesting evolutionary relationships. Segments of transposons can be amplified as satellites as reported for LTR retrotransposons, LINEs, SINEs, and DNA transposons. We analyzed a limited number of published SINE-satellite examples and found them inconclusive. Please refer to specific reviews for information about other TE origins of satellites [6, 7].

1. The PolIII/TAN (formerly known as Hirt) tandem repetitive family in the newt *Cynops pyrrhogaster* [8] contains a tRNA-like sequence and is capable of in vitro transcription. Although this ~ 360-nt tandem repeat does include a tRNA-related sequence, no similar SINEs have been identified. This species is underrepresented in sequence databases, so we cannot confirm the abundance of these tandem repeats. Thus, it can hardly represent the SINE-satellite relationship.
2. The OAX repeat in *Xenopus* has a central segment derived from tRNA and is claimed to be derived from a SINE [9]. Although this region likely originates from tRNA, its relation to a *Xenopus* SINE is yet to be confirmed. Even though these tandem repeats were designated as ‘satellite I’ [10], their repeat unit is too long to be considered a proper satellite.
3. The Rana/polIII is a highly repetitive element, accounting for up to 10% of the genomes of certain Palearctic green frogs (*Rana esculenta* group) [11]. The ~ 250 nt tandem of this element shows some similarity with tRNA (64%/64 nt) but no similarity with any known SINEs outside of this particular region.
4. Finally, the European salamanders *Hydromantes* have a Hy/polIII repetitive family with a ~ 200-nt tandem repeat [12]. However, even the origin of this sequence from tRNA is questionable (there is no box B), let alone its assignment to SINEs.

When studying Squam1 and Squam3 SINEs in reptiles [13], irregular copies were found with a few or multiple tandem duplications within a SINE sequence resembling satellites. Here, we demonstrated that the association between SINEs and tandem repeats is not occasional (at least in scaled reptiles) and analyzed different aspects of this association for three SINE families (Squam1, Squam2, and Squam3) in a variety of full-genome assemblies of scaled reptiles (Squamata). These SINEs are tRNA-related but have independent origin [13–15].

## Results

Initially, we identified repetitive elements with tandem duplications of SINE-derived sequences in the Indian cobra *Naja naja* and other species in the study of Squam3 SINEs [13]. We then conducted a systematic search for similar elements across 56 completely sequenced genomes using diverse bioinformatic approaches, including some that were custom-made for this purpose. Such SINE-derived satellites (sSat) were discovered for three SINE families, Sauria (Squam1) [14, 15], Squam2 [16], and Squam3 [13], and are referred to as sSat1, sSat2, and sSat3, respectively.

**Fig. 1:**
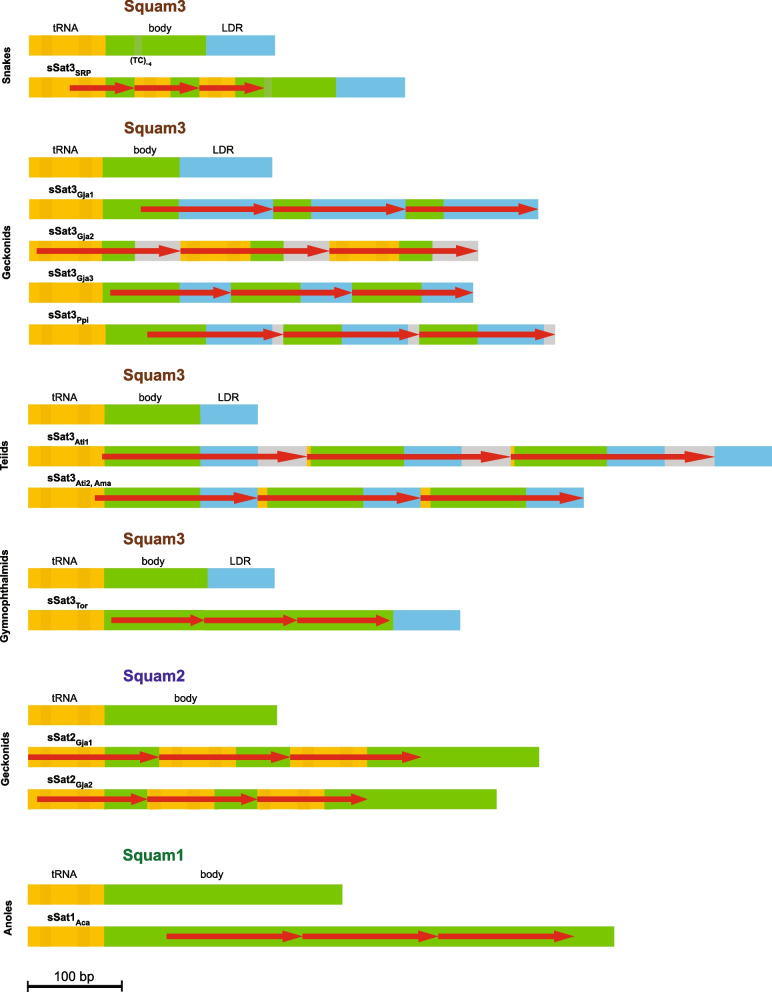
Schematic diagram of major SINE-derived satellites in squamate genomes. SINE (Squam3, Squam2, and Squam1) structure includes the tRNA-related head with the shaded promoter boxes (orange), body (green), and LINE-derived region (blue); the respective sSat regions are colored the same. Regions marked in gray have neither relation to known sequences nor to each other. The red arrows mark the tandem repeat unit (their numbers varied widely). The original SINE is specified above each panel for a given taxon (on the left). The subscript indices mark the corresponding taxon or species: SRP, Serpentes; Gja, *Gekko japonicus*; Ppi, *Paroedura picta*; Ati, *Aspidoscelis tigris*; Ama, *Aspidoscelis marmoratus*; Tor, *Tretioscincus oriximinensis*; and Aca, *Anolis carolinensis*

The sequence lengths of the three squamate SINE families range from approximately 190 to 350 nt. Notably, the sSat repeat units were around 70 to 290 nt but always shorter than the corresponding SINE, and they could cover the 5′-, middle, and/or 3′-parts of SINE (Fig. 1). The number of tandem repeat units at a specific location varied greatly, ranging from a few to hundreds. SSats with increased numbers of repeat units exhibited a uniform configuration, consistent with satellite formations. Conversely, sSats with fewer repeat units displayed greater variability in both the corresponding SINE region and sequence. The former (with at least four repeat units) were considered as nuclei for the proper satellites and given closer attention.

In our analysis of tandem sSat, the terminal 5′ (leading) and 3′ (trailing) monomers were distinguished from the internal (middle) ones. We largely considered sSat loci with at least four middle monomers (unless stated otherwise).

### Squam3-derived satellites

(sSat3) were found to be plentiful in squamates. While certain taxa lack or have marginal levels of sSat loci (disregarding Squam3 SINE numbers), five squamate taxa are known to have sSats: snakes (Serpentes), geckonids (Gekkota), teiids (Teiidae), iguanas (Iguania), and spectacled lizards (Gymnophthalmidae).

#### Squam3-derived satellites in snakes (Serpentes)

The great majority of sSats tested across 19 snake genomes exhibit high structural and sequence similarity. The tandem repeat unit covers a 3′-portion of Squam3 head, which includes the promoter box B, and a section of the SINE body (Fig. 1). The unit spans 67 nt and corresponds to positions 42–108 in Squam3C, the snake-specific Squam3 subfamily. However, two alternative tandem variants are observed in boas (Supplementary Fig. 1). The python, another primitive snake, only had 15 sSat3 loci with few tandem repeat units (mostly 2–4 monomers, at most 8) corresponding to different parts of Squam3. This is the only such example of sSat3 found in snakes; however, similar low-copy-number SINE-derived satellites were not uncommon in other squamates. We provide more detailed information on this matter in Supplementary Fig. 2.

Additionally, apart from this major tandem repeat, we encountered other variants, which contained a lesser number of repeat units and fewer loci. During our analysis, we focused on the major variants.

There was a high average similarity in the sequences found within and between sSat3 loci. As an illustration, in a sample of 45 Indian cobra loci the average sequence similarity of tandem repeat units was 89%, while the average mean similarity of Squam3 SINE sequences was 64% [13]. Meanwhile, longer loci exhibited greater homogeneity in their repeat units. For instance, the mean similarity was 82, 87, and 91% in the Indian cobra loci including 4, 5–9, and more than 10 repeat units, respectively; and the longest locus featuring 320 monomers had a similarity rate as high as 94%.

In some instances of sSat3 loci, the tandem repeat units can be separated into two or even three locus-specific subvariants characterized by a particular set of substitutions and indels. Additionally, these subvariants are frequently alternated. A manual examination of sSat3 sequences in the Indian cobra and glossy snake *Arizona elegans* showed this alternation occurring in up to 25% of lengthy loci (with a minimum of 20 repeat units) (Supplementary Fig. 3).

**Fig. 2:**
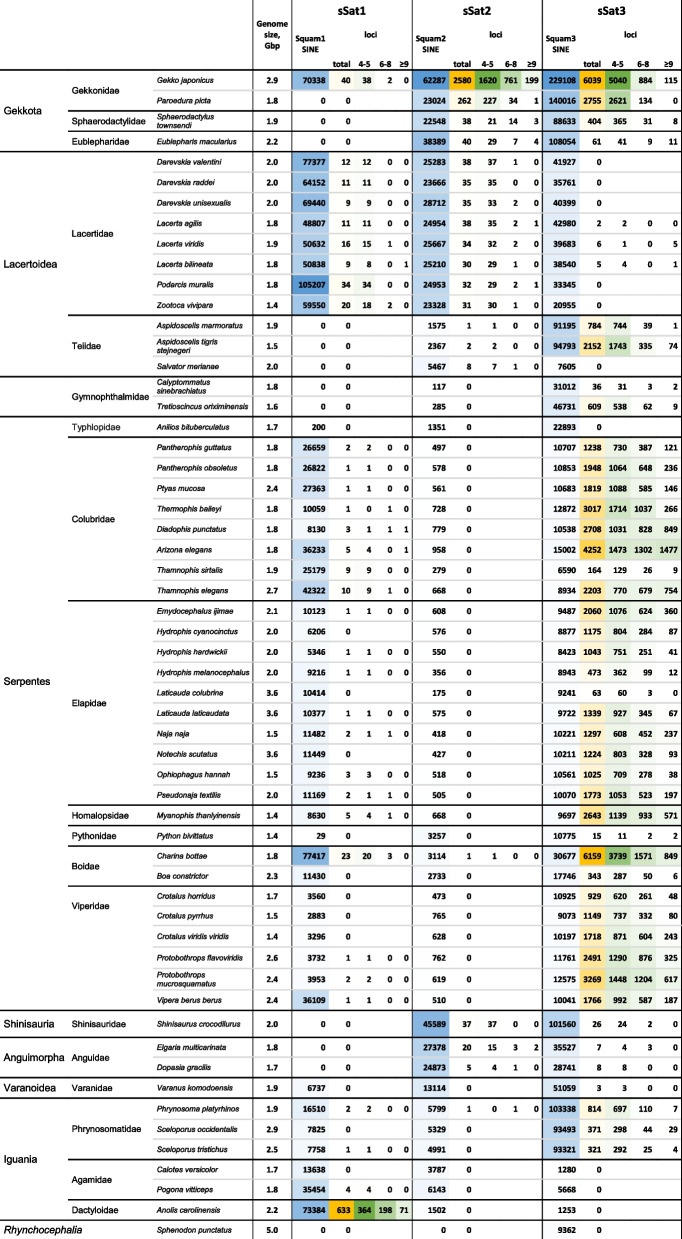
Numbers of SINE-derived satellites and the corresponding SINE loci in squamate genomes. The total number as well as that for loci with 4–5, 6–8, and more monomers are given. Relative abundance is visualized by color gradients. The only non-squamate species where Squam SINEs were found (tuatara *Sphenodon punctatus*) is given for reference. Genome sizes are given to the right of species names

The presence of sSat3 loci, which can number in the thousands in most snake species, may be less prevalent (in the hundreds and tens) in certain species or completely absent in the blind snake *Anilios bituberculatus* (Fig. 2). Typically, the number of sSat3 loci correlated with the number of Squam3 SINE; however there are a significant number of exceptions to this correlation.

Regarding the number of tandem repeat units in sSat3 loci, most snakes exhibit a relatively high proportions of lengthy sSat stretches. For instance, the loci of the Indian cobra and glossy snake contain up to 320 and nearly 950 repeat units, respectively. It is important to note that these data, particularly for the longest loci, are significantly impacted by the quality of genome assembly. Consequently, the number of sSat loci may significantly differ within the same genus (Fig. 2).

Snakes exhibit impressive consistency in the taxonomic distribution of sSat3, with no such satellite sequences found exclusively in the blind snake *Anilios bituberculatus*. The Burmese python *Python bivittatus* had as few as 15 sSat3 loci. The *Boa constrictor* had a relatively low number of sSat3 loci, while another boa species *Charina bottae* had the most sSat3 loci unearthed in this study (Fig. 2).

##### Microsatellites

The (TC)_n_ region located within the Squam3 body (beginning at position 110 in Squam3C) is identified as a microsatellite tandem repeat site, and we observed significant variation in the length of this region considerable in some SINE copies. This region concludes prior to the end of the sSat3 tandem repeat unit; accordingly, no such dinucleotide variation was observed within sSat3. We note that the tails of most SINEs of the considered families have simple repeat sequences such as A_n_, (AC_2_T_3_)_n_, (TA_3_)_n_, or (CT_2_)_n_. Such regions could be deemed as SINE-derived microsatellites; however, they were neither abundant nor widespread in squamates. Therefore, we restricted our investigation to longer sSats.

#### Squam3-derived satellites in geckos (Gekkota)

Four full-length geckos’ genomes were analyzed revealing that the identified sSat3s exhibited significantly greater structural variation compared to snakes.

The Squam3-derived satellite discovered in the Schlegel’s Japanese gecko (*Gekko japonicus*) proved to be one of the most abundant sSat3 (and sSat) in squamates (Fig. 2). SSat3_Gja_ can be partitioned into three variants, each with a tandem repeat unit corresponding to different sections of Squam3 (Fig. 1). The major repeat unit of sSat3_Gja1_ corresponds to the larger central part and the 3′-terminal part of Squam3, comprising about 130 nt. Conversely, the repeat unit of Sat3_Gja2_ corresponds to the other (5′) part of Squam3 but also includes an extra ~ 30 nt of unknown origin, totaling approximately 150 nt. The third minor sSat3_Gja3_ corresponds to the central part of Squam3 that covers most of the SINE head and half of LDR, making up 126 nt in length (Supplementary Fig. 4). The mean similarities of the repeat units are 86, 77, and 85%, respectively.

Furthermore, sSat3_Gja1_ can be categorized into three variants identifiable by 16- or 23-nt truncations at the 5′-end of the tandem repeat unit (Fig. S4). The respective proportions of these variants constitute 27, 20, and 19% of all sSat3_Gja_ loci; and the mean repeat unit similarities are 92, 85, and 87%, respectively. Noteworthily, the proportion between these variants was comparable in relatively short loci (four monomers); however, the proportion of the shortest sSat3_Gja1a_ increased with the number of monomers and became higher in loci with more than 10 monomers (Supplementary Fig. 5).

The ocelot gecko *Paroedura picta* possesses two Squam3-derived satellite variants. The major sSat3_Ppi1_ (92% of all sSat3_Ppi_ loci) resembled sSat3_Gja1_ with the exception of an additional 14-nt region of unknown origin at the repeat unit’s 5′ end (Fig. 1). On average, the tandem length was 143 nt. Despite sharing a similar structure, sSat3_Gja_ and sSat3_Ppi_ exhibit sequence differences, as depicted in Supplementary Fig. 4. The minor variant sSat3_Ppi2_ (6%) had a repeat unit that matched the tRNA portion of Squam3. It is predominantly present in short satellite loci and is exceedingly rare in longer ones. The average length of the repeat unit was 151 nt, while their similarities were 82 and 63%, respectively.

In the Townsend’s least gecko *Sphaerodactylus townsendi*, there is a noticeable decrease in the number of Squam3-derived satellites (Fig. 2) with highly diverse repeat units that preclude grouping. However, it is evident that either 5′- or 3′-terminal regions initiated this sSat3.

In the leopard gecko *Eublepharis macularius*, Squam3-derived satellite loci were rare and highly diverse indicating unsuccessful sSat3 germination.

#### Among the three teiid genomes

Analyzed, sSat3 was found in both the western *Aspidoscelis tigris* and marbled *A. marmoratus* whiptails. The *A. tigris* genome contained two variants of the repeat unit, sSat3_Ati1_ had a match with the very end of the head, the entire Squam3 body, LDR, and an additional 44 nt of unknown origin at the 3′ end (215 nt), while sSat3_Ati2_ had a slightly longer Squam3 head-related part, the entire body, and LDR (179 nt) (Supplementary Fig. 4). SSat3_Ati1_ was ~three times more abundant than sSat3_Ati2_ (48 and 29%) and this ratio remained stable in sSat3_Ati_ loci of varying lengths. In *A. marmoratus*, sSat3 corresponded to sSat3_Ati1_ but its abundance was roughly three times less despite similar numbers of Squam3 SINE (Fig. 2). The mean similarity of tandem repeat units was 97 and 84%, respectively. Both whiptail genomes showed a prevalence of relatively short tandem repeats (4–5 monomers), although the sequence similarity was higher in longer loci. SSat3 was not detected in the black and white tegu *Salvator merianae*, which matched a ~ 12-fold difference in the Squam3 abundance between these genera (Fig. 2).

#### Gymnophthalmid lizards

Exhibited relatively low numbers of sSat3 loci in the two genomes analyzed. The Oriximina lizard *Tretioscincus oriximinensis* harbored the major sSat3 variant (94% of all sSat3_Tor_ loci), which covered most of the Squam3 central region (Fig. 1). The mean similarity of sSat3_Tor_ repeat units was 92%. Minor variants covered most of the Squam3 body but their limits differed from one locus to another. This was also applicable to the gymnophthalmid *Calyptommatus sinebrachiatus*, which had a very small number of sSat3 loci (Fig. 2) with variable repeats.

#### Phrynosomatid lizards

Had a relatively low number of sSat3 satellite loci despite numerous Squam3 SINEs. All sSat3 loci within the three analyzed genomes were short and heterogeneous (Fig. 2) with a notable exception of a distinct locus found in the western fence lizard *Sceloporus occidentalis* including as much as over 1200 relatively homogeneous monomers.

### Squam2-derived satellites

(sSat2) were largely found in geckos, although minor number of loci are scattered among lizards (Lacertidae, Shinisauridae, Anguidae, and Teiidae).

#### Squam2-derived satellites in geckos

The most prevalent sSat2 was identified in *Gekko japonicus* (Fig. 2). The major sSat2_Gja1_ (79% of all sSat2_Gja_ loci) has the tandem repeat unit (139 nt) corresponding to the 3′ part of Squam2 covering the head and one-third of the body. The second variant sSat2_Gja2_ (14%) covers the same region truncated at both sides (115 nt) (Fig. 1 and Supplementary Fig. 6). The positions of tandem repeat units along the Squam2 sequence are presented in Supplementary Fig. 7. Short loci (4–6 middle monomers) with sSat2_Gja1_ were roughly six times more common than those with sSat2_Gja2_, but the proportion of sSat2_Gja2_ increased with the locus length. Most (but not all) sSat2_Gja_ loci were composed of a single monomer type. The mean similarity of sSat2_Gja1_ and sSat2_Gja2_ repeat units was 83 and 88%, respectively; however, similarity could reach 100% in the longest loci (20–60 monomers).

The *Paroedura picta* genome does not have numerous sSat2 loci (Fig. 2), most of which are short and heterogeneous. No definite variants could be identified.

#### Two other geckonid lizards

*Eublepharis macularius* and *Sphaerodactylus townsendi*, have a very low number of sSat2 loci (Fig. 2). Single relatively long loci with more than 10 monomers display uniform middle repeat units; conversely, short loci demonstrate significant heterogeneity.

#### Other squamates

(lacertids, *Shinisaurus crocodilurus*, and *Elgaria multicarinata*) where sSat2 could be found also had low sSat2 numbers. The number of monomers rarely exceeded four, and no structural patterns could be revealed.

### Squam1-derived satellites

(sSat1) could be identified in the fewest number of squamate taxa. Specifically, sSat1 was only abundant in the green anole *Anolis carolinensis* (Fig. 2). The major sSat1_Aca_ (88% of all loci) corresponds to around half of the Squam1 body (Fig. 1) but includes minor variants with relatively long central or terminal deletions. The sSat1_Aca_ repeat unit is 145 nt long (Supplementary Fig. 9) and its mean similarity is 81%. The minor variants largely confined to regions nearer to the Squam1 head.

In other taxa, sSat1 was found in low numbers of loci without a consistent pattern, although Squam1 SINE was present in high abundance in the genomes of lacertids and some other squamates (Fig. 2).

### Variation of orthologous sSat3 loci

We examined the variation of SINE-derived satellites using sSat3_SRP_ as an example in two rattlesnakes, *Crotalus pyrrhus* and *C. viridis*. We identified all pairs that contained a minimum of two sSat3_SRP_ repeat units in one genome and had matching flanking sequences in the other genome. Most loci had the same number of tandem repeat units (74%) or differed by one (14%). However, some loci varied by 10 or more repeat units (Supplementary Fig. 8A). Examination of these orthologous pairs revealed significant variation in their structure beyond the number of tandems. Some pairs consisted of only tandem fragments or included insertions of unknown origin. Many also contained stretches of N resulting from poor sequencing. Three examples are shown in Supplementary Fig. 8B. The top one represents the majority of identified loci with the same number of repeat units. The middle diagram depicts a relatively rare example with distinct tandem numbers (note the shortened rightmost repeat unit in both genomes). The bottom diagram shows an uncommon variant with both Squam3 SINE termini and several tandem repeats in between. This pilot analysis showcased a promising approach and should be expanded to incorporate additional genomes and a wider variety of species. However, further research is required to explore this matter fully.

### Chromosomal localization of SINE-derived satellites

Satellites are a component of heterochromatin, specifically, the pericentromeric and subtelomeric domains [1], in contrast to microsatellites and retrotransposons, which are mostly found in euchromatin. We investigated the chromosomal localization of sSat3, the most consistent of the SINE-derived satellites, in three snake genomes assembled to near-chromosome level (Indian cobra *Naja naja*, Lataste’s viper *Vipera latastei*, and many-banded krait *Bungarus multicinctus*). The assemblies are fairly accurate but not flawless, particularly in the problematic large repetitive regions. The assemblies comprise some fragments (“chromosomes”) that are relatively long (≥100 Mbp) and a plethora of shorter ones.

Typically, sSat3 appeared in the subtelomeric areas in the best assembled chromosomes (not necessarily the largest); other fragments could have these repeats at a single end of the fragment. Most of the shorter fragments only displayed sporadic occasions of sSat3.

**Fig. 3:**
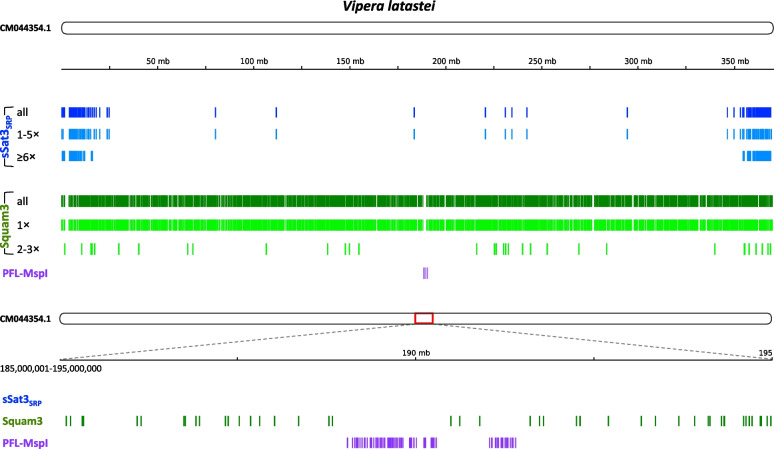
Chromosomal localization of sSat3 (blue), Squam3 SINE (green), and putative centromeric satellite PFL-MspI (purple) in the largest chromosome assembly (CM044354.1, 370 Mbp) of the Lataste’s viper *Vipera latastei*. Size fractions corresponding to 1–5 and ≥ 6 tandems for sSat3 as well as 1 and 2–3 Squam3 tandem repeats are shown below in lighter shades. The lower panel is the putative pericentromeric area (10 Mbp, 185,000,001-195,000,000) containing PFL-MspI and Squam3 but no sSat3

There is only one fragment/chromosome (in *N. naja*) that may indicate a pericentromeric localization of such repeats (Supplementary Fig. 10B). To our knowledge, only three centromeric repeats were reported in two snake species [17]. Our search for these repeats revealed only a few (0–10) such sequences in the genomes of Indian cobra and many-banded krait. However, one of them (PFL-MspI) from *Protobothrops flavoviridis* (Viperidae) proved abundant (> 1000 of loci ~ 15% of which included more than a hundred tandems) in the genome of the Lataste’s viper; their consensus sequence proved 68% identical to PFL-MspI (Supplementary Fig. 10A). Moreover, they localized to two neighboring regions in an internal part of all properly assembled chromosomes and were arranged in long tandem arrays (Fig. 3), suggesting that this PFL-MspI is a pericentromeric satellite in the Lataste’s viper as well.

We evaluated the distribution of longer and shorter stretches of tandem repeats and found a scarcity of long stretches (more than 10×) within the internal chromosome regions. This could be a result of fewer number of longer sequences. Squam3 sequences were used as a control, displaying an expected random distribution.

Recently, a highly accurate squamate genome assembly was published for the leopard gecko *Eublepharis macularius*. Telomeric termini were identifiable on certain chromosomes (Supplementary Fig. 10C). While sSat3 is less abundant than in most snakes, there is tendency to subtelomeric regions. The distribution of rare sSat2 is indeterminate.

We used the top-rated vertebrate genomic assembly (human T2T-CHM13v2.0) as a reference for visualizing the distribution of selected satellites and retrotransposons (Supplementary Fig. 10D). Generally, satellites largely localized in the (peri)centromeric regions. Specifically, α-satellites mark centromeres, while pericentromeric regions are occupied by HSATII satellites and/or (GGAAT)_n_ microsatellites, particularly in chromosomes with very long pericentromeric regions (e.g., 1, 9, and 16). The predominant SINEs and LINEs (Alu and L1) are evenly distributed throughout chromosomes but are absent from the centromeric and long pericentromeric regions.

The pattern observed for Squam3 SINE and PFL-MspI satellite was mostly replicated (Fig. 3 and Supplementary Fig. 10). However, sSat3 unequivocally exhibited subtelomeric rather than pericentromeric localization in the three snake genomes. Additionally, the CENP-B motif, a marker for specific centromeric satellites [18], was not detected.

## Discussion

SINE-derived satellites were identified based on their similarity with SINEs. In squamates, most sSats are tandem repeats with a longer terminal unit on either end (3′ or 5′) sharing similarity with the corresponding SINE region up to its terminus (Fig. 1). This pattern was common for all major sSat instances identified in this study. However, there were minor variants without such extended terminal units or with two of them corresponding to both SINE termini (as shown in the lower panel of Supplementary Fig. 8B).

Some genomes have a small number of sSat loci with only a few tandem repeat units resulting in significant variation in the corresponding SINE regions across loci (as seen in *Python bivittatus*, Supplementary Fig. 2). In contrast, other genomes have multiple and lengthy sSat loci, containing up to 1000 repeat units. These sSat loci display high homogeneity of tandem sequences both within and between loci, and this homogeneity increases with the number of repeat units per locus (Supplementary Fig. 5). This is in line with the concept of concerted evolution of satellites [1], indicating that only the abundant sSat variants undergo homogenization. However, even the genomes rich in sSats contain a few short loci with variable repeat units. Please note that most, if not all, of these numeric values are underestimated due to poor genome sequencing (which is especially conspicuous in repetitive DNA regions).

Satellites discovered come from three SINE families, namely, Sauria (Squam1), Squam2, and Squam3. Squam3 are more or less evenly distributed throughout squamates (and are even found in the tuatara of the distinct order Rhynchocephalia). Squam2 SINEs, on the other hand, are present in all squamates with varying genomic copy numbers that can differ by up to 500-fold in extreme cases. Squam1 presents a random distribution in squamates and is absent in approximately 15% of genomes tested (Fig. 2). This distribution pattern was somewhat replicated by sSats. Notably, no sSat1 was detected in the genomes lacking Squam1, including teiid (Teiidae) and spectacled (Gymnophthalmidae) lizards as well as species with a low number of SINE copies numbered in the hundreds, such as Squam2 in most snakes. Nevertheless, there are numerous genomes with a relatively high number of SINE copies that lack derived satellites, for example, Squam3 in lacertids. Likewise, no significant correlation was found between the abundance of sSats (as well as SINEs) and the genome size (Fig. 2).

The taxonomic variation of sSats was irregular. Excluding two exceptions being snakes presenting amazingly uniform sSat_SRP_ and two *Aspidoscelis* whiptails, most distant taxa possessed differing sSat repeat unit structures (Fig. 1). This pattern is typical for species-specific satellites but can also be observed in taxa of different ranks [1]. The number of sSat loci exhibits significant variation and can exceed 20 fold in representatives of the same genus (*Laticauda laticaudata* and *Laticauda colubrina*). This type of variation is not uncommon for satellites [2].

It should be noted that proper SINEs can also form tandem dimers, trimers, and so on. The frequency of tandem SINEs is generally low, but in some cases, it can be significant. For example, nearly half of Dip SINE copies in the genome of Gobi jerboa *Allactaga bullata* exist in multimers consisting of full-length Dip and/or B1 SINEs with tandems reaching up to eight [19]. At the same time, there are SINEs that amplify (i.e., are transcribed and integrated into the genome) as dimeric tandems such as the well-known *Alu* [20]; dimeric SINEs are known in vertebrates, insects, and plants [21]. In addition, SINEs with short (~ 30 nt) internal duplications were also reported [22, 23]. We found no true dimeric Squam SINEs in the genomes analyzed. The repetitive elements discussed embody a vast spectrum of structures from SINEs bearing extended internal duplications to satellite-like tandem repeats with primordial SINE sequences at either end.

The lengths of repeat units constitute a range from 150 to 180 bp or 300 to 360 bp in numerous satellites [2]. It is a prevalently accepted hypothesis that links these lengths to the DNA length wrapped around one or two nucleosomes [24]. It may be of interest in this context to note the alternating (~di and trimeric) pattern of monomers observed in the glossy snake *Arizona elegans* (Supplementary Fig. 3) and some other species, suggesting that the elongation of such satellites involves dimeric rather than monomeric units.

Most of sSat variants appear to have independent origins in different squamates. However, sSat3 in snakes, particularly in higher snakes, presents a contrasting opposite example (Supplementary Fig. 1). Another example, though less conclusive, is sSat3_Ppi_ in the gecko *Paroedura picta*, which only matches a subsubvariant sSat3_Gja1b_ in *Gekko japonicus* (Supplementary Fig. 4).

Analysis of sSats in genomes with high sSats abundance indicates that initially diverse variants tend to evolve into a few or even a single variant that dominates in terms of locus number, length, and sequence homogeneity. In the case of *Gekko japonicus*, three sSat3 subvariants are equally represented in short loci while long loci are largely composed of sSat3_Gja1_ (Supplementary Fig. 5).

Overall, certain primary duplications are more favorable as satellite targets for further amplification, while most remain as single genomic instances. The genome’s susceptibility to repeat amplification also plays an important role. For example, *Gekko japonicus* exhibits a particularly high abundance of sSat variants, and its genome is larger than that of most other squamates. However, there are other species with even larger genomes (e.g., the sea krait *Laticauda colubrina*), but they exhibit almost no sSat. The factor that makes organisms susceptible to this remains to be revealed.

According to Garrido-Ramos, different families of satellites tend to occupy the same regions of chromosomes, where heterochromatin amplification is initiated [1], mostly pericentromeric or subtelomeric regions. Our findings indicate that sSats have a clear preference for subtelomeric regions, which is more pronounced for longer loci (Fig. 3 and Supplementary Fig. 10), though they are not as abundant.

The concept of satellite emergence [2, 25] is a consistent process with three distinct steps. First, individual genomic sequences duplicate to create a “library” of short tandem repeats. Second, some of these repeats undergo further amplification, the mode of which can differ in descendant species. Finally, concerted evolution homogenizes the most successful satellite variants.

The data from this study of SINE-derived satellites in squamates generally support this concept with some specific variations. The large number of genomic SINE copies significantly enhances the likelihood of initial duplication via unequal homologous recombination [26]. Additionally, insertion into the same locus becomes much more probable.

The SINE-derived satellites identified in squamates have only reached the stage of approaching concerted evolution and are not as homogeneous as other satellites. However, the mechanisms of homogenization are more efficient for longer tandem arrays [27]; likewise, we have observed that long sSat_SRP_ loci contain more uniform repeat sequences.

We did not come across such satellites in our studies of mammalian SINEs, although we haven’t specifically checked. Therefore, it is uncertain whether these SINE-derived satellites are restricted to squamates. Nonetheless, this approach appears to have the potential to investigate the satellite evolution and possibly broader aspects of genome existence.

## Methods

Genomic data were downloaded from NCBI Genomes (https://www.ncbi.nlm.nih.gov/genome). The following assemblies were used: Schlegel’s Japanese gecko *Gekko japonicus*, Gekko_japonicus_V1.1; ocelot gecko *Paroedura picta*, Ppicta_v2.0; Townsend’s least gecko *Sphaerodactylus townsendi*, MPM_Stown_v2.3; Valentin’s lizard *Darevskia valentini*, Dval_245; sand lizard *Lacerta agilis*, rLacAgi1.pri; European green lizard *Lacerta viridis*, ASM90024590v1; western green lizard *Lacerta bilineata*, *L. bilineata* genome assembly; common wall lizard *Podarcis muralis*, PodMur_1.0; viviparous lizard *Zootoca vivipara*, UG_Zviv_1; marbled whiptail *Aspidoscelis marmoratus*, AspMar1.0; western whiptail *Aspidoscelis tigris stejnegeri* rAspTig1.0.p; Argentine black and white tegu *Salvator merianae* HLtupMer6; lesser microteiid *Calyptommatus sinebrachiatus*, HLcalSin1; Oriximina lizard *Tretioscincus oriximinensis*, HLtreOri1; prong-snouted blind snake *Anilios bituberculatus*, ASM2237905v1; corn snake *Pantherophis guttatus*, UNIGE_PanGut_3.0; western rat snake *Pantherophis obsoletus*, UNIGE_PanObs_1.0; Dhaman *Ptyas mucosa*, UNIGE_Pmuc_v1.0; Bailey’s snake *Thermophis baileyi*, DSBC_Tbai_1.0; ringneck snake *Diadophis punctatus similis*, rDiaPun1.0; glossy snake *Arizona elegans*, rAriEle1.0.p; eastern garter snake *Thamnophis sirtalis*, Thamnophis_sirtalis-6.0; western terrestrial garter snake *Thamnophis elegans*, rThaEle1.pri; turtlehead sea snake *Emydocephalus ijimae*, emyIji_1.0, Asian annulated sea snake *Hydrophis cyanocinctus*, ASM402372v1; Hardwick’s sea snake *Hydrophis hardwickii*, ASM402376v1; slender-necked sea snake *Hydrophis melanocephalus*, hydMel_1.0; yellow-lipped sea krait *Laticauda colubrine*, latCor_2.0; blue-ringed sea krait *Laticauda laticaudata*, latLat_1.0; Indian cobra *Naja naja*, NN_10x_BNG; mainland tiger snake *Notechis scutatus*, TS10Xv2-PRI; king cobra *Ophiophagus hannah*, OphHan1.0; eastern brown snake *Pseudonaja textilis*, EBS10Xv2-PRI; *Myanophis thanlyinensis*, ASM1765603v1; Burmese python *Python bivittatus*, Python_molurus_bivittatus-5.0.2; northern rubber boa *Charina bottae*, rChaBot1.0.p; timber rattlesnake *Crotalus horridus*, ASM162548v1; southwestern speckled rattlesnake *Crotalus pyrrhus*, CrotMitch1.0; prairie rattlesnake *Crotalus viridis viridis*, UTA_CroVir_3.0; habu *Protobothrops flavoviridis*, HabAm_1.0; brown spotted pitviper *Protobothrops mucrosquamatus*, P.Mucros_1.0; common viper *Vipera berus berus*, Vber.be_1.0; Chinese crocodile lizard *Shinisaurus crocodilurus*, IOZ_Scro_1.0; southern alligator lizard *Elgaria multicarinata webbii*, rElgMul1.0.p; Komodo dragon *Varanus komodoensis*, ASM479886v1; desert horned lizard *Phrynosoma platyrhinos*, MUOH_PhPlat_1.1; western fence lizard *Sceloporus occidentalis*, rSceOcc1.0.p; plateau fence lizard *Sceloporus tristichus*, ASM1680106v1; garden lizard *Calotes versicolor*, ASM2071127v1; central bearded dragon *Pogona vitticeps*, pvi1.1; and green anole *Anolis carolinensis*, AnoCar2.0. The genomes of the boa *Boa constrictor*, leopard gecko *Eublepharis macularius*, and Asian glass lizard *Dopasia gracilis* were obtained from various sources.

To refine the consensus SINE sequences in the studied genomes, we searched for close to full-length SINE copies with more than 90% length and more than 65% similarity to the consensus using the ssearch36 program from the FASTA package, followed by analysis of at least 100 randomly selected copies, and using the SubFam script. Briefly, the script partitioned a set of sequences of individual SINE loci, presorted by similarity using MAFFT [28] into groups of 100 copies, created a multiple alignment and a consensus sequence for each group, and all such consensus sequences were in turn aligned. The resulting alignment was used to estimate the diversity of SINEs. In each genome, all sequences with similarities of at least 20% of the consensus sequence length were searched for the three families (Squam1, Squam2, and Squam3). This size of the SINE fragments found allowed us to detect all genomic loci potentially containing tandemly duplicated SINE fragments. The genomic coordinates of fragments found less than 100 bp apart were merged together, and those that were longer than 500 bp after fusion were considered as candidates for containing a satellite.

Each candidate locus was analyzed using custom scripts to reveal tandemly repeated SINE-derived regions. The loci containing more than four such tandems within 100 bp from each other were considered as an sSat. The sSat monomers were counted, aligned, and comparatively analyzed between and within the loci. This made it possible to reveal tandem subvariants and to evaluate sSat diversity including the relative frequency of sSat variants in individual loci and in the whole genome.

For several snake species with relatively good genome assemblies, the chromosomal localization of the found sSat loci was analyzed in comparison with certain tandem and interspersed repeats using the Integrative Genomics Viewer (https://igv.org; IGV-Web app v.1.12.5).

Amazingly, overlapping tandem repeats such as (ATGGA)_n_, (TGGAA)_n_, etc. and their complementary variants are annotated separately by RepeatMasker; this also applies to their ~dimer (ATcGAATGGA)_n_ etc. All these were combined as (GGAAT)_n_ in Supplementary Fig. 10.

Orthologous sSat3_SRP_ loci in the genomes of the southwestern speckled rattlesnake *Crotalus pyrrhus* and prairie rattlesnake *Crotalus viridis* were analyzed by domestic bash and Perl scripts using the following pipeline. Briefly, (1) identification of sSat3_SRP_ tandem repeats (starting from two) in one genome; (2) identification of loci in the second genome with both flanking regions at a reasonable distance from each other; (3) analysis of found pairs in two genomes without flanking sequences.

### Supplementary Information

**Additional file 1 **Multiple alignment of consensus sequences of sSat3 repeat units in snakes. The Squam3 consensus sequence is given above. Subscript indices indicate the following species. Pgu, *Pantherophis guttatus*; Lco, *Laticauda colubrina*; Pob, *Pantherophis obsoletus*; Ael, *Arizona elegans*; Pte, *Pseudonaja textilis*; Cho, *Crotalus horridus*; Cvi, *Crotalus viridis viridis*; Pfl, *Protobothrops flavoviridis*; Prm, *Protobothrops mucrosquamatus*; Vbe, *Vipera berus*; Pmuc, *Ptyas mucosa*; Dpu, *Diadophis punctatus*; Tel, *Thamnophis elegans*; Tsi, *Thamnophis sirtalis*; Hcy, *Hydrophis cyanocinctus*; Hha, *Hydrophis hardwickii*; Hme, *Hydrophis melanocephalus*; Nna, *Naja naja*; Oha, *Ophiophagus hannah*; Mth, *Myanophis thanlyinensis*; Tba, *Thermophis baileyi*; Eij, *Emydocephalus ijimae*; Lla, *Laticauda laticaudata*; Nsc, *Notechis scutatus*; Cpy, *Crotalus pyrrhus*; Bco1 and Bco2, *Boa constrictor*; and Cbo, *Charina bottae*. The snake families are colored: Colubridae, yellow; Elapidae, green; Viperidae, blue; Boidae, red; and Homalopsidae, magenta.

**Additional file 2. **A. Multiple sequence alignment of consensus sequences of repeat units of Squam3-derived satellites from the Burmese python *Python bivittatus*. While all these sequences share similarity with the Squam3 consensus (upper) sequence, they correspond to different SINE regions and have little in common with each other. B. Three examples of sSat3_Pbi_ loci. The upper one (Pbi-672 in panel A) has the repeat unit covering the body of Squam3; the leading and trailing monomers also contain the 5′ and 3′ parts of Squam3, respectively. The repeat unit of the second one (Pbi-809) corresponds partially to the Squam3 head and body but has an extra 62-nt sequence of unknown origin at the 3′ end. The lower one (Pbi-558) demonstrates a highly irregular structure.

**Additional file 3. **Three examples of alternating tandem repeat units in Squam3-derived satellites in the glossy snake *Arizona elegans*. Each multiple alignment includes consecutive middle repeat units of a particular genomic locus (specified above) and the top consensus sequence. Background sequence colors mark different variants of tandem repeat units.

**Additional file 4. **Multiple alignment of consensus sequences of sSat3 in geckos, teiids, and gymnophthalmids. Names on the left indicate corresponding species. Gja, *Gekko japonicus*; Ppi, *Paroedura picta*; Ati, *Aspidoscelis tigris*; Ama, *Aspidoscelis marmoratus*; Tor, *Tretioscincus oriximinensis*. Indices after the species code indicate (sub)variants. The lizard families are colored: Gekkota, yellow; Teiidae, green; and Gymnophthalmidae, blue. Squam3 SINE consensus sequence specific for geckos and lacertids is given above.

**Additional file 5. **Distribution of three subvariants of sSat3_Gja1_ as a function of number of repeat units in the loci of the Schlegel’s Japanese gecko *Gekko japonicus*. The ordinate represents the number of repeat units in loci. The abscissa shows the proportion of each sSat3_Gja_ subvariant (A) or their absolute number (B).

**Additional file 6. **Multiple alignment of consensus sequences of sSat2 in the Schlegel’s Japanese gecko *Gekko japonicus.* The gecko-specific Squam2 consensus sequence is given above.

**Additional file 7.** Distribution of nucleotide positions of sSat2_Gja_ with 10 middle monomers along Squam2 consensus sequence. Each position of all monomers was compared against Squam2 sequence and assigned a weight of 10 for a match; 1, for mismatch, and 0 for a gap. The cumulative statistics is presented. Abscissa: Squam2 consensus position; ordinate: total position weight.

**Additional file 8. **Variation of orthologous sSat3_SRP_ loci in two snakes, southwestern speckled rattlesnake *Crotalus pyrrhus* and prairie rattlesnake *Crotalus viridis*. A. Distribution of orthologous sSat3_SRP_ loci by difference in the number of tandem repeat units. B. Examples of orthologous sSat3_SRP_ loci in *C. pyrrhus* and *C. viridis* genomes. The red arrows indicate the repeat units, while the green and blue ones correspond to the 5′ and 3′ ends of Squam3 SINE. No flanking genomic sequences are shown. Genomic affiliations are given above diagrams.

**Additional file 9. **Sequence alignment of Squam1 SINE and sSat1_Aca_ repeat unit from the green anole *Anolis carolinensis*.

**Additional file 10. **Chromosomal localization of certain genomic repeats in selected chromosomes of some squamates and human (for reference). A. Chromosomal localization of certain genomic repeats in selected chromosomes of many-banded krait *Bungarus multicinctus* and Indian cobra *Naja naja*. Blue bars correspond to sSat3, light blue bars show stretches of 1–5 and ≥ 6 tandems; and green bars show Squam3 SINE. B. Chromosomal localization of certain genomic repeats in selected chromosomes of leopard gecko *Eublepharis macularius*. Dark and light blue bars correspond to sSat3 and sSat2, respectively; dark and light green bars correspond to Squam3 and Squam2, respectively; T_2_AG_3_ are stretches of at least 1000 repeats marking telomeres. C. Chromosomal localization of certain genomic repeats in the human genome (T2T-CHM13): euchromatic interspersed retrotransposons L1 LINE (brown) and Alu SINE (green) as well as heterochromatic satellites (blue) including centromeric α-satellite (light blue) and pericentromeric HSAT2 and GGATT microsatellites (purple) as annotated by Repeat Masker (see Materials and Methods). The upper panel shows the largest chromosome 1 (248 Mbp) with an extended pericentromeric region, while the lower panel represents a typical chromosome 7 (161 Mbp).

## Data Availability

The data generated are available in the manuscript supporting files.
