# Supplementary material for: SINE-derived satellites in scaled reptiles
Source: Mob DNA. 2023 Dec 7;14:21. doi: 10.1186/s13100-023-00309-2 (PMC10702118; doi:10.1186/s13100-023-00309-2)
Supplement: Supplementary file 1 — Additional file 1 Multiple alignment of consensus sequences of sSat3 repeat units in snakes. The Squam3 consensus sequence is given above. Subscript indices indicate the following species. Pgu, Pantherophis guttatus; Lco, Laticauda colubrina; Pob, Pantherophis obsoletus; Ael, Arizona elegans; Pte, Pseudonaja textilis; Cho, Crotalus horridus; Cvi, Crotalus viridis viridis; Pfl, Protobothrops flavoviridis; Prm, Protobothrops mucrosquamatus; Vbe, Vipera berus; Pmuc, Ptyas mucosa; Dpu, Diadophis punctatus; Tel, Thamnophis elegans; Tsi, Thamnophis sirtalis; Hcy, Hydrophis cyanocinctus; Hha, Hydrophis hardwickii; Hme, Hydrophis melanocephalus; Nna, Naja naja; Oha, Ophiophagus hannah; Mth, Myanophis thanlyinensis; Tba, Thermophis baileyi; Eij, Emydocephalus ijimae; Lla, Laticauda laticaudata; Nsc, Notechis scutatus; Cpy, Crotalus pyrrhus; Bco1 and Bco2, Boa constrictor; and Cbo, Charina bottae. The snake families are colored: Colubridae, yellow; Elapidae, green; Viperidae, blue; Boidae, red; and Homalopsidae, magenta. [file 13100_2023_309_MOESM1_ESM.rtf]

                                                                                                                                                             
                 *        20         *        40         *        60         *        80         *       100         *       120         *       140         
Squam3C GAGAGCCAGTTTGGTCTAGTGGTTAAGGCACCAGGCTAGAAACCAGGAGACTGTGAGTTCTAGTCCTGCCTTAGGCATGAAAGCCAGCTGGGTGACTTTGGGCCAGTCACTCTCTCTCAGCCCAA-CCCACCTCACAGGGTTGTTGTTG
sSat3_Pgu                                        ACCAGGAGACKGTGAGTTCTAGTCCCGCCTTAGGCATGAAAGCCAGCTGGGTGACTTTGGGCCAATCAC                                       
sSat3_Lco                                        ACCAGGAGACKGTGAGTTCTAGTCCCGCCTTAGGCATGAAAGCCAGCTGGGTGACTTTGGGCCAATCAC                                       
sSat3_Pob                                        ACCAGGAGACGGTGAGTTCTAGTCCCGCCTTAGGCATGAAAGCCAGCTGGGTGACTTTGGGCCAATCAC                                       
sSat3_Ael                                        ACCAGGAGACGGTGAGTTCTAGTCCCGCCTTAGGCATGAAAGCCAGCTGGGTGACTTTGGGCCAATCAC                                      
sSat3_Pte                                        ACCAGGAGACGGTGAGTTCTAGTCCCGCCTTAGGCATGAAAGCCAGCTGGGTGACTTTGGGCCAATCAC                                       
sSat3_Cho                                        ACCAGGAGACGGTGAGTTCTAGTCCCGCCTTAGGCATGAAAGCCAGCTGGGTGACTTTGGGCCAATCAC                                       
sSat3_Cvi                                        ACCAGGAGACGGTGAGTTCTAGTCCCGCCTTAGGCATGAAAGCCAGCTGGGTGACTTTGGGCCAATCAC                                       
sSat3_Pfl                                        ACCAGGAGACGGTGAGTTCTAGTCCCGCCTTAGGCATGAAAGCCAGCTGGGTGACTTTGGGCCAATCAC                                       
sSat3_Prm                                        ACCAGGAGACGGTGAGTTCTAGTCCCGCCTTAGGCATGAAAGCCAGCTGGGTGACTTTGGGCCAATCAC                                       
sSat3_Vbe                                        ACCAGGAGACGGTGAGTTCTAGTCCCGCCTTAGGCATGAAAGCCAGCTGGGTGACTTTGGGCCAATCAC                                       
sSat3_Pmuc                                       ACCAGGAGACTGTGAGTTCTAGTCCCGCCTTAGGCATGAAAGCCAGCTGGGTGACTTTGGGCCAATCAC                                       
sSat3_Dpu                                        ACCAGGAGACTGTGAGTTCTAGTCCCGCCTTAGGCATGAAAGCCAGCTGGGTGACTTTGGGCCAATCAC                                       
sSat3_Tel                                        ACCAGGAGACTGTGAGTTCTAGTCCCGCCTTAGGCATGAAAGCCAGCTGGGTGACTTTGGGCCAATCAC                                   
sSat3_Tsi                                        ACCAGGAGACTGTGAGTTCTAGTCCCGCCTTAGGCATGAAAGCCAGCTGGGTGACTTTGGGCCAATCAC                                       
sSat3_Hcy                                        ACCAGGAGACTGTGAGTTCTAGTCCCGCCTTAGGCATGAAAGCCAGCTGGGTGACTTTGGGCCAATCAC                                       
sSat3_Hha                                        ACCAGGAGACTGTGAGTTCTAGTCCCGCCTTAGGCATGAAAGCCAGCTGGGTGACTTTGGGCCAATCAC                                       
sSat3_Hme                                        ACCAGGAGACTGTGAGTTCTAGTCCCGCCTTAGGCATGAAAGCCAGCTGGGTGACTTTGGGCCAATCAC                                       
sSat3_Nna                                        ACCAGGAGACTGTGAGTTCTAGTCCCGCCTTAGGCATGAAAGCCAGCTGGGTGACTTTGGGCCAATCAC                                       
sSat3_Oha                                        ACCAGGAGACTGTGAGTTCTAGTCCCGCCTTAGGCATGAAAGCCAGCTGGGTGACTTTGGGCCAATCAC                                       
sSat3_Mth                                        ACCAGGAGACTGTGAGTTCTAGTCCCGCCTTAGGCATGAAAGCCAGCTGGGTGACTTTGGGCCAATCAC                                       
sSat3_Tba                                        ACCAGGAGAC-GTGAGTTCTAGTCCCGCCTTAGGCATGAAAGCCAGCTGGGTGACTTTGGGCCAATCAC                                       
sSat3_Eij                                        ACCAGGAGAC-GTGAGTTCTAGTCCCGCCTTAGGCATGAAAGCCAGCTGGGTGACTTTGGGCCAATCAC                                       
sSat3_Lla                                        ACCAGGAGAC-GTGAGTTCTAGTCCCGCCTTAGGCATGAAAGCCAGCTGGGTGACTTTGGGCCAATCAC                                       
sSat3_Nsc                                        ACCAGGAGAC-GTGAGTTCTAGTCCCGCCTTAGGCATGAAAGCCAGCTGGGTGACTTTGGGCCAATCAC                                       
sSat3_Cpy                                        ACCAGGAGAC-GTGAGTTCTAGTCCCGCCTTAGGCATGAAAGCCAGCTGGGTGACTTTGGGCCAATCAC                                       
sSat3_Bco1                                                    GAGTTCTAGTCCCGCCTTAGGCATGAAAGCCGGCTGGGTGACCTTGGGCCAGTCCCTCTCTCTCAGCCCAAGAGCCAATCA-GGCGTAGTAGGA 
sSat3_Bco2                                     AAACCAGGAGACTGAGAGTTCTAGTCCCGCCTTAGGCATGAAAGCCGGCTGGGTGACCTTGGGCCAGTCCCTCTCTCTCAGCCCTAGGAAGGAGCCAATGGC        
sSat3_Cbo                                      AAACCAGGAGACTGAGAGTTCTAGTCCCGCCTTAGGCATGAAAGCCGGCTGGGTGACCTTGGGCCAGTCCCTGTCTCTCAGCCCTAGGAAGGAGCCAATGGC        
                                                                                                                                                             
                                                                                                           
        *       160         *       180         *       200         *       220         *       240        
Squam3C TGGGGAAAATAGGAGGAGGAAGGAGTATTAGATATGTTTGCCRCCTTGAGTTATTTATAAAAATAATAAAGGTGGGATAAAAATAAATAAAWAAAAAAA
sSat3_Pgu 
sSat3_Lco 
sSat3_Pob 
sSat3_Ael 
sSat3_Pte 
sSat3_Cho 
sSat3_Cvi 
sSat3_Pfl 
sSat3_Prm 
sSat3_Vbe 
sSat3_Pmuc
sSat3_Dpu 
sSat3_Tel 
sSat3_Tsi 
sSat3_Hcy 
sSat3_Hha 
sSat3_Hme 
sSat3_Nna 
sSat3_Oha 
sSat3_Mth 
sSat3_Tba 
sSat3_Eij 
sSat3_Lla 
sSat3_Nsc 
sSat3_Cpy 
sSat3_Bco1
sSat3_Bco2
sSat3_Cbo 
                                                                                                           
