# Supplementary material for: SINE-derived satellites in scaled reptiles
Source: Mob DNA. 2023 Dec 7;14:21. doi: 10.1186/s13100-023-00309-2 (PMC10702118; doi:10.1186/s13100-023-00309-2)
Supplement: Supplementary file 2 — Additional file 2. A. Multiple sequence alignment of consensus sequences of repeat units of Squam3-derived satellites from the Burmese python Python bivittatus. While all these sequences share similarity with the Squam3 consensus (upper) sequence, they correspond to different SINE regions and have little in common with each other. B. Three examples of sSat3Pbi loci. The upper one (Pbi-672 in panel A) has the repeat unit covering the body of Squam3; the leading and trailing monomers also contain the 5′ and 3′ parts of Squam3, respectively. The repeat unit of the second one (Pbi-809) corresponds partially to the Squam3 head and body but has an extra 62-nt sequence of unknown origin at the 3′ end. The lower one (Pbi-558) demonstrates a highly irregular structure. [file 13100_2023_309_MOESM2_ESM.doc]

**A**

Squam3C     GAGAGCCAGTTTGGTCTAGTGGTTAAGGCACCAGGCTAGAAACCAGGAGACTGTGAGTTCTAGTCCTGCCTTAGGCATGAAAGCCAGCTGGGTGACTTTGGGCCAGTCACTCTCTCTCAGCCCAACCCACCTCACAGGGT---TGTTGTTGTGGGGAAAATAGGAGGAGGAAGGAGTATTAGATATGTTTGCCRCCTTGAGTTATTTATAAAAATAATAAAGGTGGGATAAAAATAAATAAAWAAAAAAA
Pbi-672(4) GGCCAGTCCCTCTCTCTCAGCCCAACTCACCTCACAGGGT---TGTTGTTGTGGGGA
Pbi-783(4) GCCTTAGGCATGAAAGCCGGCTGGGGGACTTTGGGCCAGTCACTCTCTCTCAGCCCAACTCACC
Pbi-838(4) CAGGCTAGAAACCAGGAGACTGTGAGTTCTAGTCCCACCTTAGGCATGAAAGCTGGCTGGGTGACTTTGGGCCAGTCACTCTCTCTCAGCCCACGT
Pbi-001(10) TGGGTGACTTTGGGCCAGTCACTCTCCCTCAGCCCAACCCACCTCACAGGGT---TGTTGTTTGGGGAA
Pbi-558(4) TAGAAACCAGGAGACCCTGAGTTCTAGTCCTTCCTTAGGCATG-AAGCYAGCTGGGKGACTTTGGRCCAGTCYCTCTCTCTCAGCCCAAGAG
Pbi-945(6) CAGTGTGGTCCAGTGGGGAAGGTGCTGGACTAGAAACTGGGAGACCGTGAGTTCTAGTCCTGCCTTAGGCACA-AAGCCAGCTGGGTGACYTTGGGCCAGTCMCTCTCTCTCAGCCCAAAGGW
Pbi-809(4) TGGTATAGAGGTTAAGGCACCAGGCTAGAAACCAGGAGACCGTGAGTTCTAGTCCCACCTTAGGCACG-AAGCCAGCTGGGTGACCTTGGGCCAGTCCTTTTCTCTCTGGCTGGGGCATTCTGGGAGTTGAAGTCCACACATCTTAAAGTTGT----CAAGGTTGAGAAACAC
Pbi-545(4) GTCCCGTTTTAGGCACA-AAGCCAGCTGGGTGACCTTGGGCCAGTCCCTCTCTCTCAGCCCTAGGAAGAAGGCAAGG
Pbi-706(4) GGGAAAATAGGAGGA---GGAAGGAGTATTAGGTATGTTCACCACCTTGAGTTATTTATAAAAAAATAGTTGTGG
Pbi-291(4) CTCTCTCAGCCCAACCCACCTCACAGGGT---GGTGGTTATGGGGAAAATAGGTGACTAGGTCAGTCC
Pbi-114(4) TAGTGGTGAAGGTGCTGGCCTAGAAACCAGGAGACTGCAAATTCTAGTCCTGCCTTAGGCGTGAAAGCCAGTTGGGTGACTTTGGGCCG------------------------TCTCACAGGGTTGTTGTTGTTGTTGGGAAAACAGTA---GGCAGGAGTATTAGTA-TGTTTGCTGCCTTGAGTTGACACACACACAAACACACATATAGTGAGATAAACAT
Pbi-807(4) CTGAGTGACTTGGGGCCAGTCAGTCTCTC--AGCCCAACCTACTTAGCAGGGT---TGT-GTTGTAGGGAAAATGGAGGAAGGATAGCTGTGAACAGCTCA
Pbi-542(5) CCCAACAACCTCACAGGGT---TGTTGTTGTGGGCAAAATAGGA---GGCAGGAGTATTAGATATGCACAG
Pbi-431(9) GAGAGCCAGTTTGGTCTAGTGGTTAAGGCTTCAGGCTAGAAGCCTGG-GATTGTGAATTAT-----CCCCCTAGACATGAAAGGCGGCTGGTTGACGNTNGGCCAGTTTCNCTCTCTCNGNCCAGGAGCCAAGCTNCTCTGACNAACGNTAGGAAGAAAANAAAATGCTATTCTGGAACAAGAATAGTCAA
Pbi-004(6) AGATCCAGTTTGGTCTAGTGGTTAAGGCACCAGGTTAGAAACCAGAAGACTGTGAGTTCTAGTCCTGCCCTAGGCATGGAAG--------------------CCGTCACTCTCTCTCAGCCCATGA----------------TGAGGCTTGGGCGACAAGCTAGACCAAT

**B**

**Pbi-672:**
 * 20 * 40 * 60 * 80 * 100 * 120 * 140 * 160
Squam3C GAGAGCCAGTTTGGTCTAGTGGTTAAGGCACCAGGCTAGAAACCAGGAGACTGTGAGTTCTAGTCCTGCCTTAGGCATGAAAGCCAGCTGGGTGACTTTGGGCCAGTCACTCTCTCTCAGCCCAACCCACCTCACAGGGTTGTTGTTGTGGGGAAAATAG
KE956294.1:43908-44298:1-150   GAGCCAGTTTGATCTAGTGGTTACGGCACCAGGCTAGAAAGCAGAGGAC--TGAGTTCTAGTCCCGCCTTAGGCACGAAAGCCGCCTGGGTGACTTTGGGCCAGTCCCTCTCTCTCAGCCCAACTCACCTCACAGGGTTGTTGTTGTGGGGA      
KE956294.1:43908-44298:151-204                                                                                                     GGCCAGTCCCTCTCTCTCAGCCCAACTCACCTCACAGGGTTGTTGTTGTGGGGA      
KE956294.1:43908-44298:205-258                                                                                                     GGCCAGTCCCTCTCTCTCAGCCCAACTCACCTCACAGGGTTGTTGTTGTGGGGA      
KE956294.1:43908-44298:259-390                                                                                                     GGCCAGTCCCTCTCTCTCAGCCCAACTCACCTCACAGGGTTGTTGTTGTGGGGAAAATAG

 * 180 * 200 * 220 * 240
Squam3C GAGGAGGAAGGAGTATTAGATATGTTTGCCRCCTTGAGTTATTTATAAAAATAATAAAGGTGGGATAAAAATAAATAAAWAAAAAAA
KE956294.1:43908-44298:0-150                                                                                        
KE956294.1:43908-44298:151-204                                                                                        
KE956294.1:43908-44298:204-258                                                                                        
KE956294.1:43908-44298:259-390 GAGGAGGAAGAAACATTAGGTATGTTTGCCACCTTGAGTTATTTATAAAAATAATAAAGGTGGGGTAATTAT               

**Pbi-809:**

* 20 * 40 * 60 * 80 * 100 * 120 * 140 * 160
Squam3C GAGAGCCAGTTTGGTCTAGTGGTTAAGGCACCAGGCTAGAAACCAGGAGACTGTGAGTTCTAGTCCTGCCTTAGGCATGAAAGCCAGCTGGGTGACTTTGGGCCAGTCACTCTCTCTCAGCCCAACCCACCTCACAGGGTTGTTGTTGTGGGGAAAATAG
KE958017.1:10246-10826:1-135                                            CAGGC-ACTGTGAGTTCTAGTCCCACCTTAGGCACG-AAGCCAGCTGGGTGACCTTGGGCCAGTCCTTTTCTCTCTGGCTGGGGCATTCTGGGAGTTGAAGTCCACACATCTTAAAG
KE958017.1:10246-10826:136-303            TGGTATAGAGGTTAAGGCACCAGGCTAGAAACCAGGAGGCCGTGAGTTCTAGTCCCACCTTAGGCACG-AAGCCAGCTGGGTGACCTTGGGCCAGTCCTTTTCTCTCTGGCTGGGGCATTCTGGGAGTTGAAGTCCACACATCTTAAAG
KE958017.1:10246-10826:304-471            TGGTATAGAGGTTAAGGCACCAGGCTAGAAACCAGGAGACCGTGAGTTCTAGTCCCACCTTAGGCACG-AAGCCAGCTGGGTGACCTTGGGCCAGTCCTTTTCTCTCTGGCTGGGGCATTCTGGGAGTTGAAGTCCACACATCTTAAAG
KE958017.1:10246-10826:472-580            TGGTATAGAGGTTAAGGCACCAGGCTAGAAACCAGGAGACCGTGAGTTCTAGTCCCGCCTAAGGCACA-AGGCCAGCTGGGTGACCTTGGGCCAGTCCCTCTCTCTCAGC                                       


 * 180 * 200 * 220 * 240
Squam3C GAGGAGGAAGGAGTATTAGATATGTTTGCCRCCTTGAGTTATTTATAAAAATAATAAAGGTGGGATAAAAATAAATAAAWAAAAAAA
KE958017.1:10246-10826:1-135 TTGTCAAGGTTGAGAAACAC                                                                   
KE958017.1:10246-10826:136-303 TTGTCAAGGTTGAGAAACAC                                                                   
KE958017.1:10246-10826:304-471 TTGTCAAGGTTGAGAAACAC                                                                   
KE958017.1:10246-10826:472-580                                                                                        

**Pbi-558:**
 * 20 * 40 * 60 * 80 * 100 * 120 * 140 * 160
Squam3C GAGAGCCAGTTTGGTCTAGTGGTT-AAGGCACCAGGCTAGAAACCAGGAGACTGTGAGTTCTAGTCCTGCCTTAGGCATGAAAGCCAGCTGGGTG-ACTTTGGGCCAGTCACTCTCTCTC--AGCCCAA--CCCACCTCACAGGGTTGTTGTTGTGGGGA
KE955406.1:63703-64169:1-142 GAGAGCCAATTTGGTACGATGGTTGAAGGGAGCAGGCTAAAAACCAAGAGACTGTGAGTTCTAGTCCTCCGTCAGGCATGGAAGTCAGCTGGGTG-ACTTTGGACCCATCTCTCCCTCTC--AGCTTAAGAGCCAGGTGGTAGAT               
KE955406.1:63703-64169:143-282                                      TAGAAACTAGGAGACCCTGAGTTATAGTTCATCCTTA------GAAGCTTCCTGGGGG-ACACTGGGCCAGGCCTTCTCTC----AGCCCAAGAGTCAGTGTGATCTACTATGGAAGGAGCTG
KE955406.1:63703-64169:283-348                                                                 TCCCTCCTTAACCATAAAAGCTCTCTGGAGGTACTGTGGACCAGCCCCTCTCTCTC--AGCCCAAGAG                            
KE955406.1:63703-64169:349-466       CAGTGTGGTGTAGTGGTTACAGGTGGTGGATTAGAAGCCAGGAGACCCTGAGTTCTAGTCCTGCCTTGGGCAC-GAAACCAGTTGGGTG-ACCTT-GGCCAGTCTCTCTCTCTCTTAGCCC                                 

 * 180 * 200 * 220 * 240 *
Squam3C AAATAGGAGGAGGAAGGAGTATTAGATATGTTTGCCRCCTTGAGTTATTTATAAAAATAATAAAGGTGGGATAAAAATAAATAAAWAAAAAAA
KE955406.1:63703-64169:1-142                                                                                              
KE955406.1:63703-64169:143-282 GACCAGGGGTGGGGAGATCCATCTTCCA                                                                 
KE955406.1:63703-64169:283-348                                                                                              
KE955406.1:63703-64169:349-466                                                                                              
