# Supplementary material for: SINE-derived satellites in scaled reptiles
Source: Mob DNA. 2023 Dec 7;14:21. doi: 10.1186/s13100-023-00309-2 (PMC10702118; doi:10.1186/s13100-023-00309-2)
Supplement: Supplementary file 3 — Additional file 3. Three examples of alternating tandem repeat units in Squam3-derived satellites in the glossy snake Arizona elegans. Each multiple alignment includes consecutive middle repeat units of a particular genomic locus (specified above) and the top consensus sequence. Background sequence colors mark different variants of tandem repeat units. [file 13100_2023_309_MOESM3_ESM.pdf]

JAKOOK010019927.1:14956-16503(-)

```
YAGGAGACTGGGAGTTCTAGTTCCsC--CTTAGGCATGAAAGGCAGCTGGGTGACTTTGGGCCAATCAC
      ..................A.--.........................................
C...T.T.A..............TG.--.....A...A....C.....A...AG.....A....GATG.
T........CT..........C..C.C-........C................................
C.....T.A..............TG.--.....A...A....C.....A...AG.....A.....ATG.
T........CT..........C..C.C-.........................................
C.....T.G..............TG.--.....A...A....C.....A...AG.....A....GATG.
T........CT..........C..C.C-........C................................
C.....T.G..............TG.--.....A...A....C.....A...AG.....A....GATG.
T........CT..........C..C.C-........C................................
C.....T.G..............TG.--.....A...A....C.....A...AG.....A.....ATG.
T........CT..........C..C.C-.........................................
C.....T.G..............TG.--.....A...A....C.....A...AG.....A....GATG.
T........CT..........C..C.C-........C................................
C.....T.G..............TG.--.....A...A....C.....A...AG.....A.....ATG.
T........CT..........C..C.CG........C................................
C.....T.G..............TG.--.....A...A....C.....A...AG.....A....GATG.
T........CT..........C..C.C-........C................................
C.....T.G..............TG.--.....A...A....C.....A...AG.....A.....ATG.
T........CT.T...T....C..C.C-........C................................
C.....T.G..............TG.--.....A...A....C.....A...AG.....A.....ATG.
T........CT..........C..C.G-........C................................
C.....T.G..............TG.--.....A...A....C.....A...AG.....A.....ATG.
T........CT.T...T....C..C.C-........C................................
```

JAKOOK010005013.1:4-2501(-)

```
ACCAGGAGATGGTGAGTTCTAGTCCTGCCyTTGGCATGAAAGtCGACTGGGTGACTTTAGGCCaATC
............G..........T.CA..A.A...G......C.TG..-.........GA...G...
..TC.ACA..................A..T.A.CA.......CT.G.........A...........
.........C...................C............CT..................TTT..
..T..........................T..T......C........A.A.......GA.......
.........C...................C............-....................TT..
..T..........................T..T......C........A.A.......GA.......
.........C...................C............-...................TTT..
..T..........................T..T......C........A.A.......GA.......
.........C...................C............-...................TTT..
..T..........................T..T......C........A.A.......GA.......
.........C...................C............-....................TT..
..T..........................T..T......C........A.A.......GA.......
.........C...................C............-...................TTT..
..T..........................T..T......C........A.A.......GA.......
.........C...................C............-....................TT..
..T..........................T..T......C........A.A.......GA.......
.........C...................C............-....................TT..
..T..........................T..T......C........A.A.......GA.......
.........C...................C............-....................TT..
..T..........................T..T......C........A.A.......GA.......
.........C...................C............-...................TTT..
..T..........................T..T......C........A.A.......GA.......
.........C...................C............-....................TT..
..T..........................T..T......C........A.A.......GA.......
.........C...................C............-T..................TTT..
..T..........................T..T......C........A.A.......GA.......
.........C...................C............-....................TT..
..T..........................T..T......C........A.A.......GA.......
.........C...................C............-....................TT..
..T..........................T..T......C........A.A.......GA.......
.........C...................C............CT...................TT..
..T..........................T..T......C........A.A.......GA.......
.........C...................C............-...................TTT..
..T..........................T..T......C........A.A.......GA.......
.........C...................C............-...................TTT..
..T..........................T..T......C........A.A.......GA.......
.........C...................C............-...................
```

JAKOOK010003824.1:1668-6032(-)

```
ACCAGGAGACGGgGAGTTCTAGTCCCGCCTTAGGTATGAAAGC-----TGGCTGGGTGACTTTGGGCCAATC
..T...G..TA...............A..A...AA..A...A.-----CA.........T.C..........
............A..A......C..TA.....A..T......T-----..C...AA...............T
...G.....A.AA.............A................CAGCTCA......................
......T...A...............A..A...AA..A...A.-----CA.........T.C..........
...............T................A..T......T-----..C...AA...............T
...G.....A.AT...........TT..T.....C........-----.T.A..............T.....
.........T..A..A.......T.......TA.......G..-----..C...AA........A.......
............T.............A.T..............CAGCTCA......................
..........A...............A..A...AA..A...A.-----CA.........T.C..........
...............A................A..T......T-----..C...AA...........G...T
...G.....A.AT...........TT.TT.....C........-----...A..............T....T
............A..A.......T.......TA..........-----..C...AA........A.......
.........A.AA.............A................CAGCTCA......................
......T...A...............A..A...AA..A...A.-----CA.........T.C..........
...............T................A..T......T-----..C...AA..........TG...T
...G.....A.AT...........TT..T.....C........-----...A..............T.....
............A..A.......T.T.....TA.......G..-----..C...AA........A.......
............T.............A.T..............CAGCTCA......................
..........A...............A..A...AA..A...A.-----CA.........T.C..........
...............A................A..T......T-----..C...AA...........G...T
...G.....A.AT...........TT.TT.....C........-----...A..............T....T
............A..A.......T.......TA..........-----..C...AA........A.......
.........A.AA.............A................CAGCTCA......................
......T...A...............A..A...AA..A...A.-----CA.........T.C..........
...............T................A..T......T-----..C...AA...............T
...G.....A.AA..........T..A................CAGCTCA......................
......T...A...............A..A...AA..A...A.-----CA.........T.C..........
...............T................A..T......T-----..C...AA...............T
...G.....A.AA..........T..A................CAGCTCA......................
..........A...............A..A...AA..A...A.-----CA.........T.C..........
...............T................A..T......T-----..C...AA..........TG...T
...G.....A.AT...........TT..T.....C........-----...A..............T.....
............A..A.......T.T.....TA.......G..-----..C...AA........A.......
............T.............A.T..............CAGCTCA......................
..........A...............A..A...AA..A...A.-----CA.........T.C..........
...............A................A..T......T-----..C...AA...........G...T
...G.....A.AT...........TT.TT.....C........-----...A..............T....T
............A..A.......T.......TA..........-----..C...AA........A.......
.........A.AA.............A................CAGCTCA......................
......T...A...............A..A...AA..A...A.-----CA.........T.C..........
...............T................A..T......T-----..C...AA...............T
...G.....A.AA..........T..A................CAGCTCA......................
......T...A...............A..A...AA..A...A.-----CA.........T.C..........
...............T................A..T......T-----..C...AA...............T
...G.....A.AA..........T..A................CAGCTCA......................
..........A...............A..A...AA..A...A.-----CA.........T.C..........
...............T................A..T......T-----..C...AA..........TG...T
...G.....A.AT...........TT..T.....C........-----...A..............T.....
............A..A.......T.T.....TA.......G..-----..C...AA........A.......
............T.............A.T..............CAGCTCA......................
..........A...............A..A...AA..A...A.-----CA.........T.C..........
...............A................A..T......T-----..C...AA...........G...T
...G.....A.AT...........TT.TT.....C........-----...A..............T....T
............A..A.......T.......TA..........-----..C...AA........A.......
.........A.AA.............A................CAGCTCA......................
......T...A...............A..A...AA..A...A.-----CA.........T.C..........
...............T................A..T......T-----..C...AA...............T
...G.....A.AA..........T..A................CAGCTCA......................
......T...A...............A..A...AA..A...A.-----CA.........T.C..........
...............T................A..T......T-----..C...AA...............T
...G.....A.AT...........TT..T.....C........-----...A..............T.....
............A..A.......T.T.....TA..........-----..C...AA.............T..
```
