# Supplementary material for: SINE-derived satellites in scaled reptiles
Source: Mob DNA. 2023 Dec 7;14:21. doi: 10.1186/s13100-023-00309-2 (PMC10702118; doi:10.1186/s13100-023-00309-2)
Supplement: Supplementary file 4 — Additional file 4. Multiple alignment of consensus sequences of sSat3 in geckos, teiids, and gymnophthalmids. Names on the left indicate corresponding species. Gja, Gekko japonicus; Ppi, Paroedura picta; Ati, Aspidoscelis tigris; Ama, Aspidoscelis marmoratus; Tor, Tretioscincus oriximinensis. Indices after the species code indicate (sub)variants. The lizard families are colored: Gekkota, yellow; Teiidae, green; and Gymnophthalmidae, blue. Squam3 SINE consensus sequence specific for geckos and lacertids is given above. [file 13100_2023_309_MOESM4_ESM.rtf]

                                                                                                                                                                             
                     *        20         *        40         *        60         *        80         *       100         *       120         *       140         *       160 
Squam3B     GAGAGCCAGTGTGGTGTAGTGGTTAAGAGCGGTAGACTCGTAATCT-GGAGAACCGGGTTCGCGTCTCCGCTCCTCCACATGCAG-CTGCTGGGTGACCTTGGGCTAGTCACACTTCT--TCTGAAGTCTCTCAGCCCCACTCACCTC-ACAGAGTGTTTG
sSat3_Gja1a -----------------------------------------------------------------------------------------------------------------------------------------------ACCTC-ACAGGGTGTCTG
sSat3_Gja1c ------------------------------------------------------------------------------------------------------------------------TCAGAGCTCTCTCAGCCCCACCTACCTC-ACAGGGTGTCTG
sSat3_Gja1b -------------------------------------------------------------------------------------------------------------------------------TCTCTCAGCCCCACCTACCTC-ACAGGGTGTCTG
sSat3_Ppi1  -------------------------------------------------------------------------------------------------------------------------------TCTCTCAGCCTCACCTCCCTC-ACAGGGTGTCTG
sSat3_Gja2  ----GCCAGTTTGGTGTAGTGGTTAAGTGC-GCGGACTC-TAATCTGGGAGAACCGGGTTTGATTCCCCACTCCTCCACTTGCACCTGGCTGGATGACCTTGGGCAAGCCATAGTTCTGATAAGAGCTGTTCTTGAAAAAGAGCAGTTTCTGTCAAGAGC-
sSat3_Ppi2  -AGAGCCAGCTTGGTGTAGTGGTTAGGAGT-GCGGACTTCTAATCT-GGCGAGCCGGGTTTGATTCCCCGCTCCCCCACATGCAGCCAGCTGGGTGACCTTGGGCTCGCCACAGCACTG-ATAAAGCTGTTCTGACCGAGCAGTAATATCAGGGC------
sSat3_Gja3  --------------------------------------------------------------------------------------CTGCTGGGTGACCTTGGGCCAGTCACAGTTCTC-TCAGAACTCTCTCAGCCCCACCTGCCTC-ACAAGGTGCCTG
sSat3_Ati1  ----------------------------------------------------------------------------------AAGCTTGCTAGGTGGCTATGGGCTAGTCACAGCTCTCTTCTGAGCTCTCACAGCCCCACCTACCTC-ACAGGGTGTCTG
sSat3_Ati2  -------------------------------------------------------------------------TCCTCCATGAAGCCAGCTGGGTGACCTTGGGCTAGTCACAGCTCTCTTCTGAGCTCTCACAGCCCCACCTACCTC-ACAGGGTGTCTG
sSat3_Ama   -------------------------------------------------------------------------TCCTCCATGAAGCCAGCTGGGTGACCTTGGGCTAGTCACAGCTCTCTTCTGAGCTCTCACAGCCCCACCTACCTC-ACAGGGTGTCTG
sSat3-Tor   ----------------------------------------------------------------------------------------GCTGGGTGACCTTGGGCCAGTCACAGTTCT--TTAGAACTCTCTCAGCCCCACCTA-CTCCACAGGGTGTCTG
                                                                                                                                                                             
                                                                                                                                                         
                    *       180         *       200         *       220         *       240         *       260         *       280         *       300  
Squam3B     TTGTGGGG-GAGGAAGGGAAAGGAGAATGTTAGCCGCTTTGAGACTCCTTCGGGTAGTGAT-AAAGCGGGATATCAAATCCAAACTCTTCTTCTTCTT-------------------------------------------
sSat3_Gja1a TTGTGGGGAGGGGAAGGGAAAGGAGTTTGTAAGCCGCTCTGAGACTCCTTTGGGTAGTGA--AGGGCGGGGTAT-AAATCCAATCTCTTCTTCTTCTTCTTT---------------------------------------
sSat3_Gja1c TTGTGGGGAGGGGAAGGGAAAGGAGTTTGTAAGCCGCTCTGAGACTCCTTTGGGTAGTGA--AGGGCGGGGTAT-AAATCCAATCTCTTCTTCTTCTTCT-----------------------------------------
sSat3_Gja1b TTGTGGGGAGGGGAAGGGAAAGGAGTTTGTAAGCCGCTCTGAGACTCCTTTGGGTAGTGA--AGGGCGGGGTAT-AAATCCAATCTCTTCTTCTTCTTCTT----------------------------------------
sSat3_Ppi1  TTGTGGGGAGAGGAAAGGGAAGGCGANTGTAAGCCGCTTTGAGACTCCTTCGGGTAGAGA--AAAGCGGCATAT-AAGAACCAACTCTTCTTCTTCTTCAGTAATATCAGGGC----------------------------
sSat3_Gja2  ---------------------------------------------------------------------------------------------------------------------------------------------
sSat3_Ppi2  ---------------------------------------------------------------------------------------------------------------------------------------------
sSat3_Gja3  TTGTGGGGAGAGGAAGGGAAGGC-GATTGTAAGCCGCTTTGAGACACATGAGGC---------------------------------------------------------------------------------------
sSat3_Ati1  TTGTGGGGGGAAGAACAGAAA---GTTTGTCAGCCGCTTTGAGTCCCCTTAAATGGGAGAGAAAACCGGGGTAG-AAAAGCCATTGCTGCTTCTGCTTCTTCTAGGGATTAATCCAGCTTCAGTCCACTTTCACATGAGCC
sSat3_Ati2  TTGTGGGGAGAAGAACAGAAG---GTTTGTCAGCCGCTTTGAGTCCCCTTAAATGGGTGAGAAAAGCGGGGTAG-AAAAGCCAACTCTTCTTCTTC---------------------------------------------
sSat3_Ama   TTGTGGGGAGAAGAACAGAAG---GTTTGTCAGCCGCTTTGAGTCCCCTTAAATGGGTGAGAAAAGCGGGGTAG-AAAAGCCAACTCTTCTTCTTC---------------------------------------------
sSat3-Tor   TTGTGGAGAAAGCAACAAAAG---CTATGT---------------------------------------------------------------------------------------------------------------
                                                                                                                                                         
