# Supplementary figures and images for: SINE-derived satellites in scaled reptiles
Source: Mob DNA. 2023 Dec 7;14:21. doi: 10.1186/s13100-023-00309-2 (PMC10702118; doi:10.1186/s13100-023-00309-2)

A

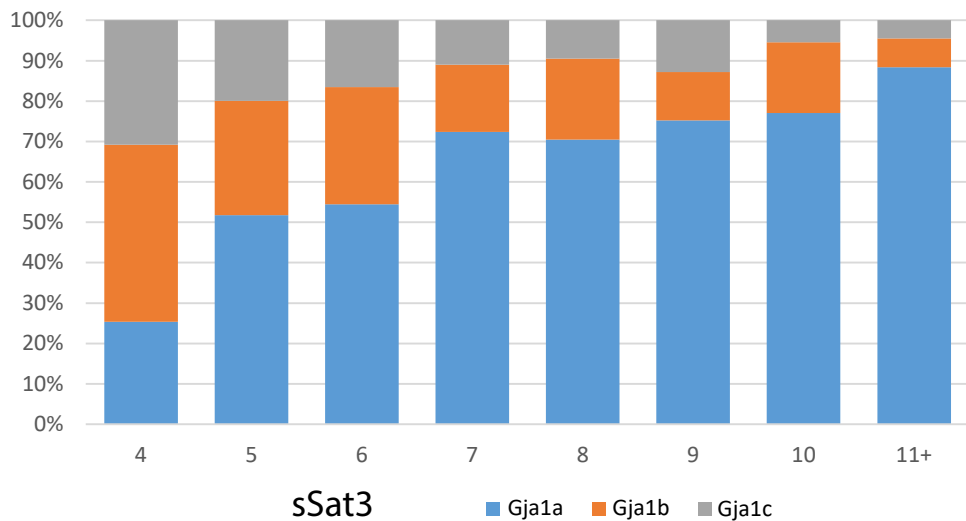

B

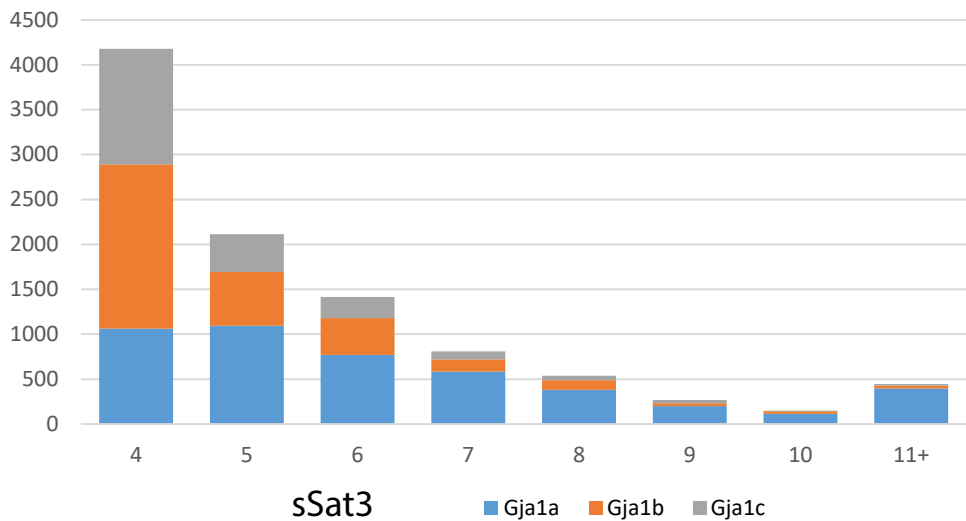

Supplement: Supplementary file 5 — Additional file 5. Distribution of three subvariants of sSat3Gja1 as a function of number of repeat units in the loci of the Schlegel’s Japanese gecko Gekko japonicus. The ordinate represents the number of repeat units in loci. The abscissa shows the proportion of each sSat3Gja subvariant (A) or their absolute number (B). [file 13100_2023_309_MOESM5_ESM.pdf]

sSat2<sub>Gja</sub>

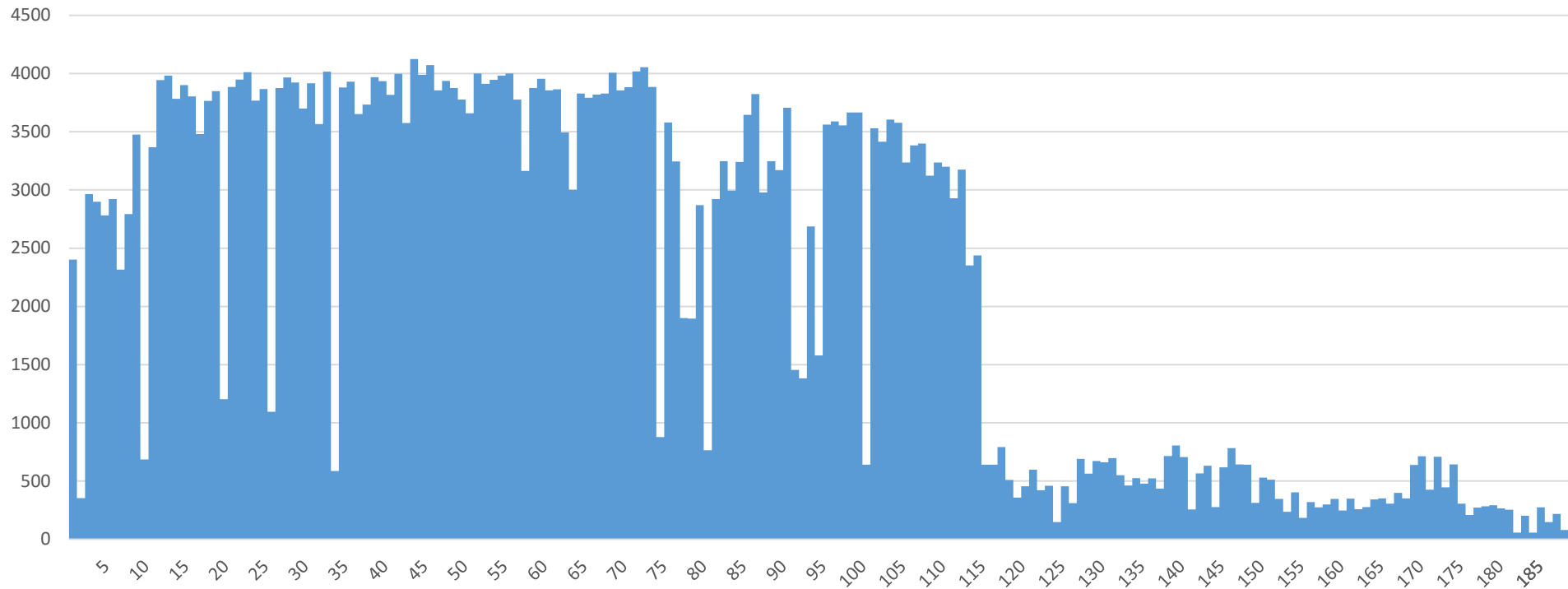

Supplement: Supplementary file 7 — Additional file 7. Distribution of nucleotide positions of sSat2Gja with 10 middle monomers along Squam2 consensus sequence. Each position of all monomers was compared against Squam2 sequence and assigned a weight of 10 for a match; 1, for mismatch, and 0 for a gap. The cumulative statistics is presented. Abscissa: Squam2 consensus position; ordinate: total position weight. [file 13100_2023_309_MOESM7_ESM.pdf]

**A.**

| **Difference between number of repeat units** | **Total  number** |
| --- | --- |
| 0 | 1653 |
| 1 | 321 |
| 2 | 126 |
| 3 | 66 |
| 4 | 32 |
| 5 | 21 |
| 6 | 19 |
| 7-9 | 6 |
| 10-15 | 4 |

**B.**
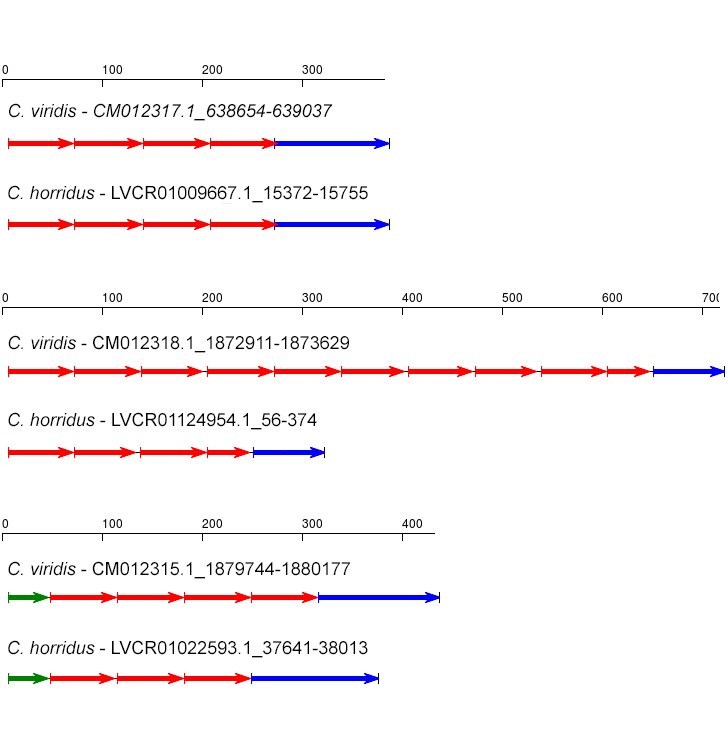

Supplement: Supplementary file 8 — Additional file 8. Variation of orthologous sSat3SRP loci in two snakes, southwestern speckled rattlesnake Crotalus pyrrhus and prairie rattlesnake Crotalus viridis. A. Distribution of orthologous sSat3SRP loci by difference in the number of tandem repeat units. B. Examples of orthologous sSat3SRP loci in C. pyrrhus and C. viridis genomes. The red arrows indicate the repeat units, while the green and blue ones correspond to the 5′ and 3′ ends of Squam3 SINE. No flanking genomic sequences are shown. Genomic affiliations are given above diagrams. [file 13100_2023_309_MOESM8_ESM.doc]
