# Supplementary material for: SINE-derived satellites in scaled reptiles
Source: Mob DNA. 2023 Dec 7;14:21. doi: 10.1186/s13100-023-00309-2 (PMC10702118; doi:10.1186/s13100-023-00309-2)
Supplement: Supplementary file 6 — Additional file 6. Multiple alignment of consensus sequences of sSat2 in the Schlegel’s Japanese gecko Gekko japonicus. The gecko-specific Squam2 consensus sequence is given above. [file 13100_2023_309_MOESM6_ESM.rtf]

                                                                                                                                                               
                    *        20         *        40         *        60         *        80         *       100         *       120         *       140        
Squam2        GGGCTGTGGCTCAGTGGTAGAGCATCTGCTTGGCATGCAGAAGGTCCCAGGTTCAATCCCTGGCATCTCCAGTTAAAA-GGAC-AGGCAG-AGGTGATGTGAAAGACCTCTG-CCTGAGACCCTGGAGAGCCGCTGCCAGTCGAG
sSat2-Gja1 GAGGGGCTGTGGCTCAGTGGCAGAGCCTCTGCTTGGCATGCAGAAGGTCCCAGGTTCAATCCCCGGCATCTCCAGTTAAAAAGGACCAGGCAGGAGGTGATGGGAAAGACCTCCG-CCTGAGACCCTGGAGAGCTGTAAA        
sSat2-Gja2            GCTCAGTGGCAGAGCCTCTGCTTGGCATGCAGAAGGTCCCAGGTTCAATCCCCGGCATCTCCAGTT-AAAAGGACCAGGCAGGAGGTGATGAGAAAGACCT-TGACCTGAGACCCTG                    
                                                                                                                                                               
                                                                        
            *       160         *       180         *       200         
Squam2     TAGACAATACTGACCTTGATGGACCAATGGTCTGATTCAGTATAAGGCAGCTTCATGTGTT
sSat2-Gja1                                                              
sSat2-Gja2                                                              
                                                                        
