# Supplementary material for: SINE-derived satellites in scaled reptiles
Source: Mob DNA. 2023 Dec 7;14:21. doi: 10.1186/s13100-023-00309-2 (PMC10702118; doi:10.1186/s13100-023-00309-2)
Supplement: Supplementary file 9 — Additional file 9. Sequence alignment of Squam1 SINE and sSat1Aca repeat unit from the green anole Anolis carolinensis. [file 13100_2023_309_MOESM9_ESM.doc]

10 20 30 40 50 60 70 80 90 100

Squam1 ggagcccccggtggcgcagtgtgttaaagcgctgagctgctgaacttgcagaccgaaaggtcccaggttcaaATCCGGGGAGCGGAGTGAGCGCCCGCTG

110 120 130 140 150 160 170 180 190 200

Squam1 TTAGCCCCAGCTTCTGCCAACCTAGCAGTTCGAAAACATGCAAATGTGAGTAGATCAATAGGTACCGCTCCGGCGGGAAGGTAACGGCGCTCCATGCAGT

::::::::::::::::::::::: ::::::::::::::::::::::::::::::::::

sSat1_Aca_  AATGTGAGTAGATCAATAGGTACTGCTCCGGCGGGAAGGTAACGGCGCTCCATGCAGT

10 20 30 40 50

210 220 230 240 250 260 270 280 290

Squam1 CATGCC---GGCCACATGACCTTGGAGGTGTCTACGGACAACGCCGGCTCTTCGGCTTAGAAATGGAGATGAGCACCAACCCCCAGAGTCAGACACGACT

:::::: ::::::::::::::::::::::::::::::::::::::::::: :::::::::::::::::::::::::::::::::::

sSat1_Aca_ CATGCCAGTGGCCACATGACCTTGGAGGTGTCTACGGACAACGCCGGCTCTT-GGCTTAGAAATGGAGATGAGCACCAACCCCCAGAG

60 70 80 90 100 110 120 130 140

300 310 320

Squam1 GGACTTAATGTCAGGGGAAAACCTTTACCTTT
