# Supplementary material for: SINE-derived satellites in scaled reptiles
Source: Mob DNA. 2023 Dec 7;14:21. doi: 10.1186/s13100-023-00309-2 (PMC10702118; doi:10.1186/s13100-023-00309-2)
Supplement: Supplementary file 10 — Additional file 10. Chromosomal localization of certain genomic repeats in selected chromosomes of some squamates and human (for reference). A. Chromosomal localization of certain genomic repeats in selected chromosomes of many-banded krait Bungarus multicinctus and Indian cobra Naja naja. Blue bars correspond to sSat3, light blue bars show stretches of 1–5 and ≥ 6 tandems; and green bars show Squam3 SINE. B. Chromosomal localization of certain genomic repeats in selected chromosomes of leopard gecko Eublepharis macularius. Dark and light blue bars correspond to sSat3 and sSat2, respectively; dark and light green bars correspond to Squam3 and Squam2, respectively; T2AG3 are stretches of at least 1000 repeats marking telomeres. C. Chromosomal localization of certain genomic repeats in the human genome (T2T-CHM13): euchromatic interspersed retrotransposons L1 LINE (brown) and Alu SINE (green) as well as heterochromatic satellites (blue) including centromeric α-satellite (light blue) and pericentromeric HSAT2 and GGATT microsatellites (purple) as annotated by Repeat Masker (see Materials and Methods). The upper panel shows the largest chromosome 1 (248 Mbp) with an extended pericentromeric region, while the lower panel represents a typical chromosome 7 (161 Mbp). [file 13100_2023_309_MOESM10_ESM.ppt]

## Slide 1
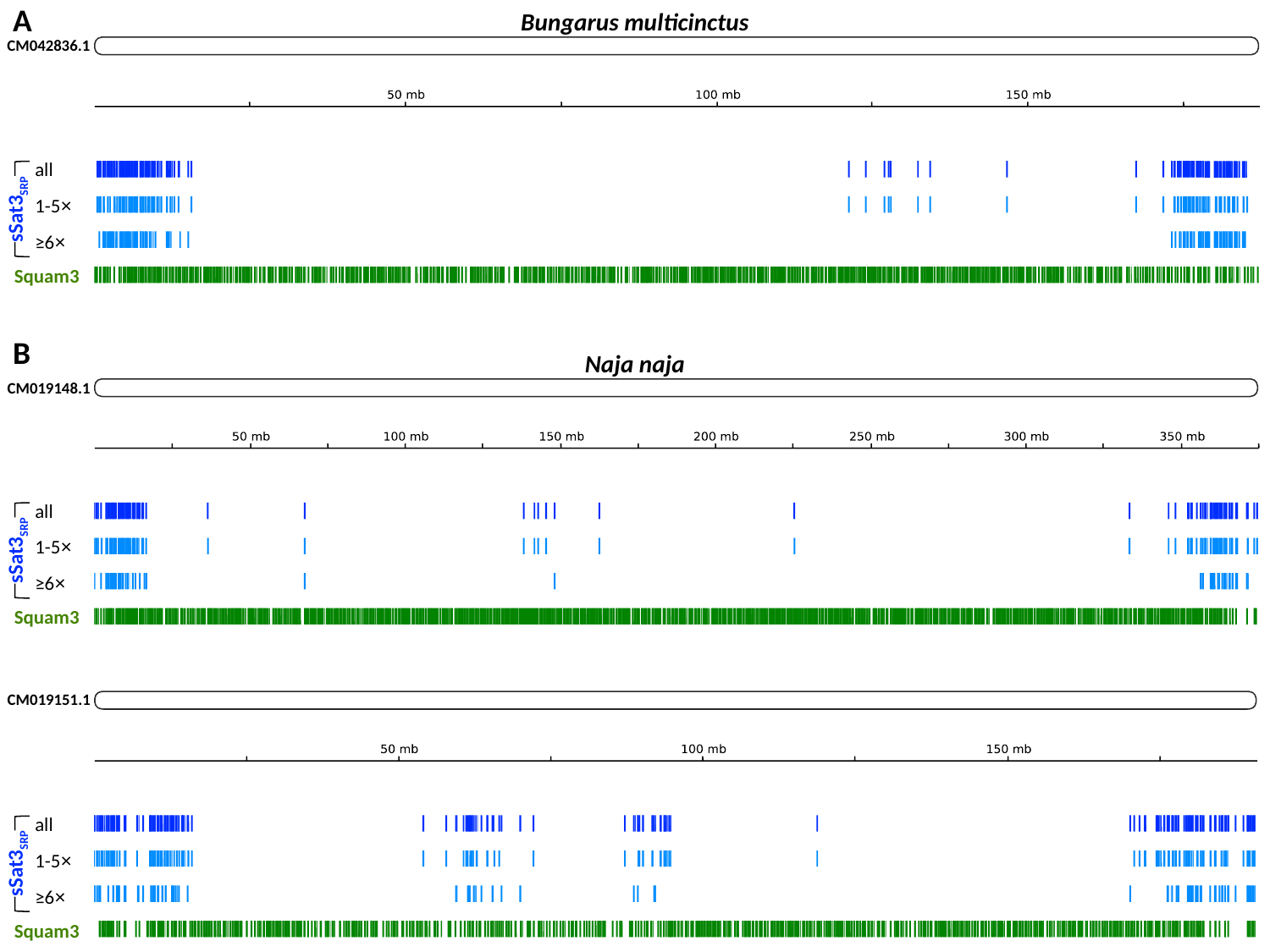

A
Bungarus multicinctus
CM042836.1
all
sSat3SRP
1-5×
≥6×
Squam3
B
Naja naja
CM019148.1
all
sSat3SRP
1-5×
≥6×
Squam3
CM019151.1
all
sSat3SRP
1-5×
≥6×
Squam3

## Slide 2
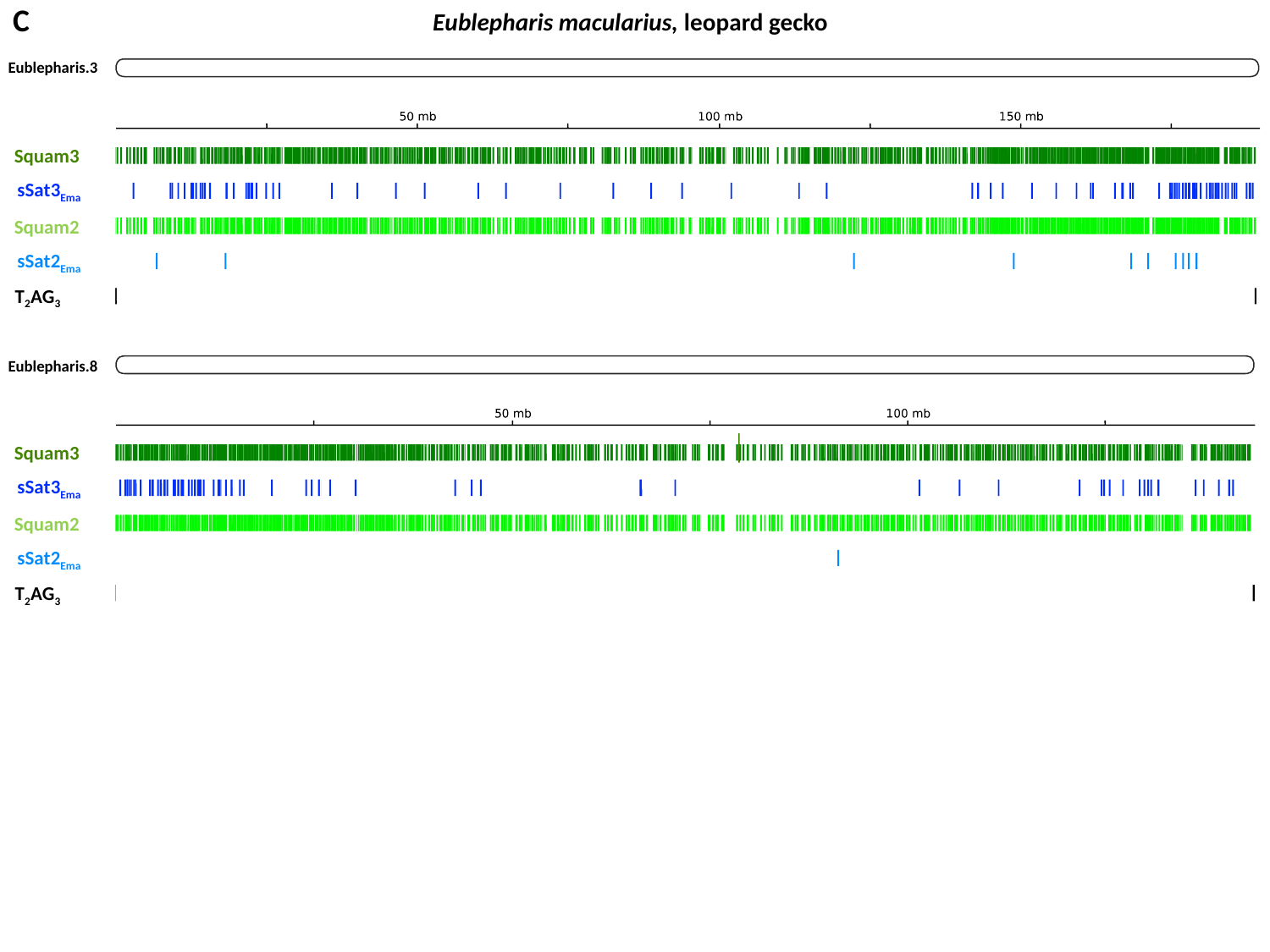

C
Eublepharis macularius, leopard gecko
Eublepharis.3
Squam3
sSat3Ema
Squam2
sSat2Ema
T2AG3
Eublepharis.8
Squam3
sSat3Ema
Squam2
sSat2Ema
T2AG3

## Slide 3
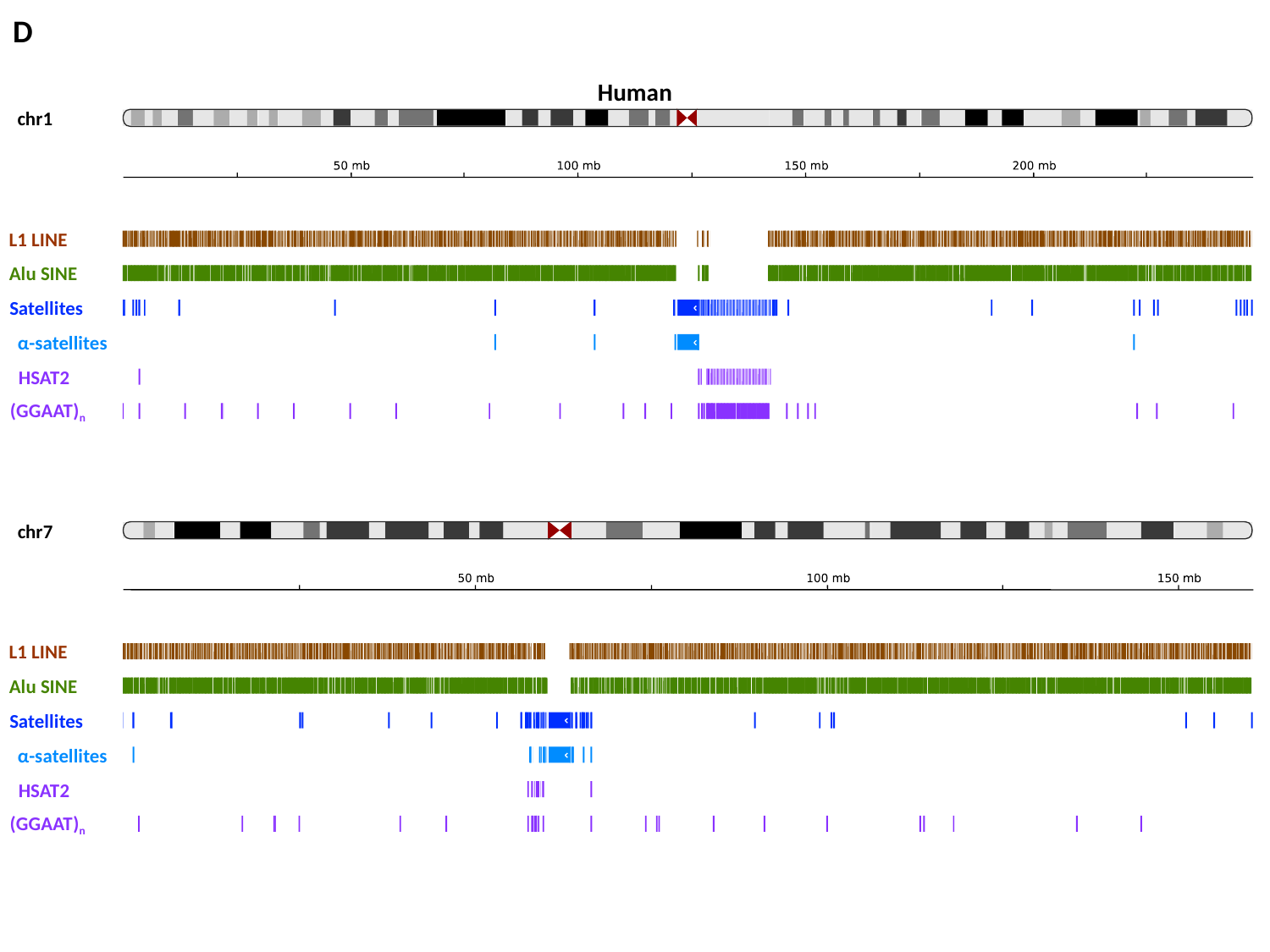

D
Human
chr1
L1 LINE
Alu SINE
Satellites
α-satellites
HSAT2
(GGAAT)n
chr7
L1 LINE
Alu SINE
Satellites
α-satellites
HSAT2
(GGAAT)n
